# Regulatory roles of an sRNA derived from the 5´ UTR and sequence internal to *lap*A in *Pseudomonas aeruginosa* PAO1

**DOI:** 10.1128/spectrum.01303-24

**Published:** 2025-04-22

**Authors:** Xiaojuan Tan, Jingjing Xiao, Qianqian Liu, Ting Yang, Dandan Feng, Ruyi Zheng, Liping Luo, Xi Cheng, Dongsheng Du, Minghui Li, Jinwei Zhou, Guoping Zhu

**Affiliations:** 1 Anhui Provincial Key Laboratory of Molecular Enzymology and Mechanism of Major Diseases, College of Life Sciences, Anhui Normal University, Wuhu, Anhui, China; 2 School of Food and Biological Engineering, Xuzhou University of Technology 117819, Xuzhou, Jiangsu, China; Beijing Institute of Genomics, Chinese Academy of Sciences, Beijing, China

**Keywords:** *Pseudomonas aeruginosa*, sRNA LapS, alkaline phosphatase LapA, virulence factors, biofilms

## Abstract

Several key virulence factors of *Pseudomonas aeruginosa* are regulated by quorum-sensing systems, small noncoding RNAs (sRNAs), and environmental stress, leading to a high mortality rate. Our previous studies indicated that the alkaline phosphatase LapA regulated *P. aeruginosa* PAO1 biofilm formation in a chronic wound model established with *ex vivo* porcine skin explants. Notably, one particular sRNA located upstream of the *lapA* gene was highly expressed in the model. Therefore, the sRNA was further characterized via northern blotting and rapid amplification of cDNA ends. The results revealed that the sRNA we named LapS is 197 nucleotides in length and is derived from the 5´ UTR and sequence internal to the *lapA* gene. Next, LapS mutation, overexpression, and complementation strains were constructed from the PAO1 strain, and phenotypic experiments associated with *lapA* were performed and compared with those of the Δ*lapA* and wild-type strains. The results indicated that LapS is involved in regulating swarming motility, rhamnolipid and alkaline phosphatase production, *las/rhl* quorum-sensing systems, and biofilm formation by controlling the level of *lapA* mRNA under phosphate-depleted conditions. Therefore, this LapS-*lapA* signaling cascade is beneficial for balancing the virulence regulation of *P. aeruginosa*. Additionally, an *in vitro* study indicated that LapS directly and post-transcriptionally regulated at least one unlinked gene, *putA*, which encodes bifunctional proline dehydrogenase, a virulence factor of *P. aeruginosa*. Collectively, our findings reveal a previously unstudied regulatory sRNA and advance the understanding of the roles of sRNAs in the pathogenicity of *P. aeruginosa*.

**IMPORTANCE:**

*Pseudomonas aeruginosa* is a common nosocomial pathogen that contains hundreds of virulence factors regulated by quorum-sensing systems and environmental stress. Small noncoding RNAs (sRNAs) involved in virulence regulation have been identified in *P. aeruginosa*. Recently, several potential sRNAs were identified in *P. aeruginosa* using transcriptome sequencing. However, some of these novel sRNAs have been functionally characterized. In this study, a previously uncharacterized sRNA, LapS, in *P. aeruginosa* PAO1 was identified as a novel sRNA. LapS is involved in regulating swarming motility, rhamnolipid production, and alkaline phosphatase production during phosphate-depleted stress by controlling the level of *lapA* mRNA. Furthermore, LapS deletion also reduced the mortality rate of *Caenorhabditis elegans* in a fast-kill assay. Additionally, LapS directly suppressed PutA, a virulence factor of *P. aeruginosa*. This study highlights the role of LapS in modulating *P. aeruginosa* virulence during phosphate-depleted stress.

## INTRODUCTION

*Pseudomonas aeruginosa* is a major nosocomial pathogen that causes serious infections in a wide range of patients, including those with burns, surgical wounds, lung diseases, or immunocompromised diseases (1). Presently, *P. aeruginosa* ranks among the top five pathogens, along with *Escherichia coli*, *Klebsiella pneumoniae*, *Staphylococcus aureus*, and *Acinetobacter baumannii*, which are found in clinical bacteremia and have high mortality rates. The pathogenicity of *P. aeruginosa* is attributed mainly to the presence of various virulence factors, such as phenazines, elastase, rhamnolipids, motility, and hemolytic ability, which play crucial roles in the development of *P. aeruginosa* infections (2–5). Importantly, *P. aeruginosa* can easily form biofilms, causing many persistent and noninvasive human infections, including chronic wound infection, cystic fibrosis, and medical device-associated infections (6, 7).

Previous studies have shown that bacterial virulence is modulated by the regulatory networks of quorum-sensing (QS) systems and environmental stress (8, 9). *P. aeruginosa* possesses at least three known QS systems, namely, *las*, *rhl*, and *pqs*, that control the expression of more than 300 genes involved in virulence factor production and biofilm formation (10, 11). *las* and *rhl* are N-acylated homoserine lactone (AHL)-signaling systems. In the *las* system, N-(3-oxo-dodecanoyl)-L-homoserine lactone (3-oxo-C12-HSL), synthesized by the autoinducer synthase LasI, interacts with the LasR receptor to activate virulence factors, such as LasB elastase, LasA protease, exotoxin A, and alkaline protease (12). Furthermore, in the *rhl* system, N-butanoyl-L-homoserine lactone (C4-HSL), produced by the autoinducer synthase RhlI, interacts with RhlR to induce the production of other virulence factors, including rhamnolipid, pyocyanin, chitinase, and hydrogen cyanide (11–13). Notably, 2-heptyl-3-hydroxy-4-quinolone is the third primary QS signal in *P. aeruginosa* and functions as a signaling molecule by binding to its cognate receptor PqsR to induce pyocyanin production (12, 13).

Environmental stress, such as phosphate depletion, can promote the production of virulence factors, allowing *P. aeruginosa* to cause acute or chronic infections (5, 14). Phosphate is essential for all living cells as an essential component of energy molecules, including ATP, nucleic acids, phospholipids in membranes, and other biomolecules; substantial depletion of phosphate has been reported after surgical injury, which can significantly increase the virulence of *P. aeruginosa* (4, 15, 16). Previous studies by our group and others have indicated that phosphate limitation reduces C4-HSL production but increases rhamnolipid production and swarming motility (17, 18). Furthermore, phosphate depletion also increases hemolysis, elastase activity, and pyocyanin production in *P. aeruginosa* (9, 19). Moreover, phosphate depletion also increases the virulence of *P. aeruginosa* to *Caenorhabditis elegans* in fast-kill and slow-kill assays (20).

Recently, many studies have shown that small noncoding RNAs (sRNAs) are involved in bacterial pathogenesis, including QS and virulence regulation (21, 22). Although several potential intergenic sRNAs have been identified in *P. aeruginosa* via transcriptome sequencing (RNA-seq) (23), few of these novel sRNAs have been functionally characterized. Recently characterized sRNAs have focused mainly on their roles in QS regulation. The *P. aeruginosa* sRNA RhlS located at the 5´ UTR of *rhlI* regulates C4-HSL production via the activation of *rhlI* translation (22). It also transcriptionally regulates the unlinked gene *fpvA*, which encodes a siderophore pyoverdine receptor. The sRNAs PhrS and PrrF modulate the *pqs* system by regulating PqsR and AntR, respectively (24, 25). The sRNA ReaL targets *pqsC* mRNA to increase PQS synthesis (26). The sRNA PrrH regulates elastase production, swimming and swarming motility, rhamnolipid production, and biofilm formation in *P. aeruginosa* (27). Additionally, PrrH regulates the hemolysis and oxidative resistance of *P. aeruginosa* during bloodstream infection (25).

In this work, to investigate a novel sRNA in *P. aeruginosa* that regulates virulence production and biofilm formation in chronic wounds, RNA-seq data were reanalyzed for *P. aeruginosa* PAO1 biofilms in an *ex vivo* wound model of porcine skin explants, which are in a phosphate-depletion environment (28). One of those screened sRNAs, LapS, was highly upregulated in biofilms, and its expression changes were verified via quantitative reverse transcriptase polymerase chain reaction (qRT-PCR). The predicted sRNA LapS is located upstream of *lapA* within the Hxc-T2SS operon. In this operon, *lapA* encodes an alkaline phosphatase, and the Hxc-T2SS is used to transport the alkaline phosphatase LapA (29). In our previous study, we reported that LapA can perform additional functions in regulating *P. aeruginosa* virulence under phosphate-depletion conditions and biofilm formation in chronic wounds (18). However, the role of LapS has rarely been studied, although it is located within the same operon as *lapA*. The sRNA LapS was thus selected for further characterization via northern blotting and rapid amplification of cDNA ends (RACE) experiments in this study. Subsequently, genotypic and phenotypic characterization of the *lap*S gene-deficient and overexpression strains revealed significant roles of LapS in controlling *lapA* expression, alkaline phosphatase production, rhamnolipid production, swarming motility, and virulence in *C. elegans* under phosphate-depleted conditions. Furthermore, it also regulated biofilm formation in chronic wounds. Additionally, our study indicated that LapS directly targeted one unlinked gene, *putA*, a virulence factor in *P. aeruginosa*. Therefore, the sRNA LapS is a novel sRNA that affects diverse virulence in *P. aeruginosa* PAO1.

## RESULTS

### Identification of sRNA candidates in PAO1 biofilms that cause chronic wound infection via RNA-seq analysis

On the basis of our previous RNA-seq data, we found that *lapA*, which encodes an alkaline phosphatase, was highly expressed when *P. aeruginosa* PAO1 formed biofilms in a chronic wound model established with *ex vivo* porcine skin explants (28). We subsequently experimentally demonstrated that the *lapA* gene plays a crucial role in the virulence and biofilm formation of *P. aeruginosa* under phosphate-depleted conditions (18). In this study, RNA-seq data were further analyzed to investigate the effects of potential factors, in addition to *lapA*, on *P. aeruginosa* virulence production and biofilm formation, such as sRNAs, in chronic wounds. First, 434 sRNAs were predicted via manual curation after Rockhopper prediction (Table S1), a software package specifically designed for small RNA and transcriptome analysis of bacterial RNA-seq data (30). Next, the differentially expressed sRNAs in the biofilms were identified via the R package DEseq2 and compared with those in planktonic cells. A total of 102 sRNAs were identified as differentially expressed in biofilms, 67 of which were elevated (Table S2). Interestingly, one of these predicted sRNAs—designated sRNA0078 with a length of 101 nt, which is located between bp 747369 and bp 747469 in the PAO1 genome, and the 5´ UTR of the *lapA* gene (PA0688)—was most highly upregulated in the mature biofilm. qRT-PCR assays revealed that the expression of sRNA0078 was greater throughout the whole biofilm development cycle than in the planktonic state (Fig. S1A). Moreover, we found that sRNA0078 is not one of 29 annotated sRNAs of PAO1 on the NCBI website (https://www.ncbi.nlm.nih.gov/gene/?term=ncRNA+PAO1). We chose to focus on sRNA0078 for two reasons. One is that the sRNA is located in the 5´ UTR of *lapA* (Fig. S1B), which could regulate the transcription or translation of *lapA*; the other is that highly expressed sRNA0078 might be associated with the virulence and biofilm formation of *P. aeruginosa* under phosphate-depletion conditions. Notably, the newly identified sRNA0078 should be further investigated by northern blotting and RACE analyses.

### sRNA LapS is derived from the 5´ UTR and sequence internal to *lapA*

**Fig 1:**
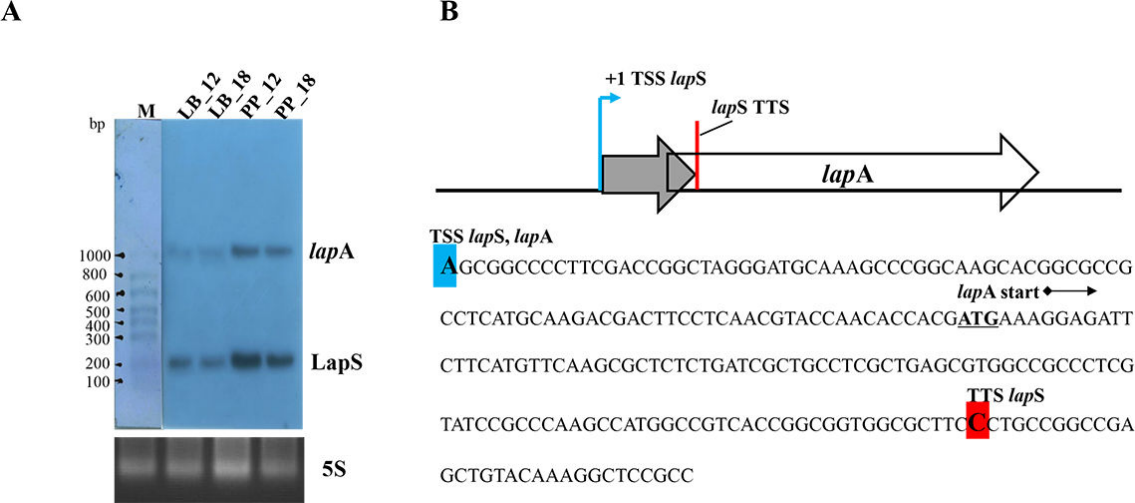
Expression of LapS under phosphate-depleted and -rich conditions. (**A**) Northern blot analysis of LapS. The WT strain was grown in phosphate-depleted (PP) or phosphate-rich (LB) media for 12 or 18 h, respectively. The cells were harvested, and RNA was extracted. For total RNA, 10 µg was analyzed via northern blotting, and 5S rRNA was used as a loading control. (**B**) Schematic and sequence of *lapS-lapA*. The blue arrow indicates the +1 site of transcription, the blue bar indicates the transcription start sites (TSSs) of LapS and *lapA*, and the red bar indicates the transcription termination site (TTS) of LapS.

Because sRNA0078 was highly expressed in PAO1 biofilms formed in ex vivo porcine skin explants, which are in a phosphate-depleted environment, we hypothesized that this sRNA could be highly expressed in phosphate-depleted medium but expressed at lower levels under phosphate-rich conditions. To verify this hypothesis, the expression of sRNA0078 was further investigated using northern blotting analyses under phosphate-depleted and phosphate-rich conditions. As shown in Fig. 1A, when PAO1 was cultured in proteose-peptone (PP) medium for 12 h, sRNA0078 was highly expressed but expressed at relatively low levels in lysogeny broth (LB) medium. In addition, northern blot analyses revealed that the size of the sRNA was ~200 bp, and the sequencing results revealed that the sRNA was located between bp 747363 and bp 747559 in the PAO1 genome, which is longer than the size predicted by the software. Importantly, there was another band at about 1,000 bp in the northern blot image (Fig. 1A). Therefore, we hypothesized that the sRNA would be transcribed together with the *lapA* gene, which might be a 5´ UTR sRNA of *lapA* similar to the sRNA RhlS in the 5´ UTR of *rhlI* in *P. aeruginosa* or similar to other sRNAs derived from the 5´ UTR and sequences internal to open reading frames in *E. coli* and *Borrelia burgdorferi* (22, 31, 32); therefore, we named this sRNA LapS (LapA-associated sRNA).

To test the above hypothesis, we mapped the 5´ ends of both LapS and *lapA* with 5´ rapid amplification of cDNA ends (RACE) and the 3´ end of LapS with 3´ RACE assays and found that the transcription start sites (TSSs) of LapS and *lapA* appeared to be one or the same, corresponding to the −107 position of *lapA*. The transcription termination site (TTS) of LapS corresponded to the +90 position of *lapA* (Fig. 1B). By using the boundaries defined by the TSS and TTS, we inferred that LapS is 197 nucleotides in length and that the size of this transcript is consistent with the size of the LapS band observed via northern blot analysis (Fig. 1A). Therefore, LapS is derived from the 5´ UTR and sequence internal to the *lapA* gene. Collectively, our data suggest that under phosphate-depletion conditions, *lapA* can be transcribed from a single *lapA* promoter into the following two isoforms: the long isoform encoding full-length *lapA* mRNA and the short isoform LapS, which was also observed via northern blot analysis.

### LapS controls the level of *lapA* mRNA under phosphate-depleted conditions

**Fig 2:**
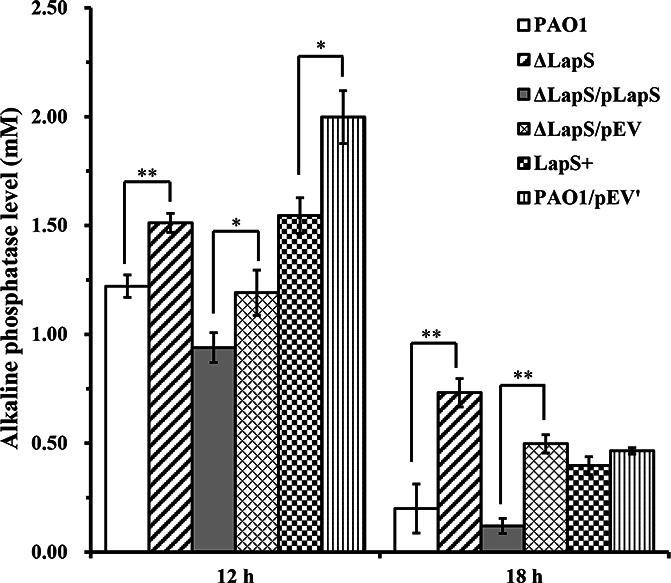
Alkaline phosphatase levels measured in the supernatants from *P. aeruginosa* PAO1, ΔLapS, LapS^+^, and complementation strains under phosphate-depleted conditions. The amount of alkaline phosphatase was defined as µmoles of *p*-nitrophenol liberated from *p*-nitrophenyl phosphate at a specific time point. The data are shown as the mean ± standard error of the mean of at least three independent experiments. *, *P* < 0.05; **, *P* < 0.01; ***, *P* < 0.001.

The LapS mutation strain was established through the homologous recombination method, and its overexpression strain was constructed from the wild-type strain PAO1 containing the pUCP18-LapS plasmid. The WT, ΔLapS, LapS^+^, and complementation strains were cultured overnight in LB medium and subsequently inoculated in fresh PP medium. The bacterial growth curves, including those for the early exponential phase, mid-exponential phase, and early stationary phase, were not different (Fig. S2). Our previous study revealed that compared with the WT strain, the Δ*lapA* strain exhibited only 26.1% total alkaline phosphatase activity at the early stationary phase (12 h), and it continuously produced alkaline phosphatase under phosphate-depletion conditions, whereas the WT strain produced very little alkaline phosphatase at 18 h. In contrast, neither the Δ*lapA* strain nor the WT strain produced alkaline phosphatase under phosphate-rich conditions (18). Therefore, in this study, alkaline phosphatase activity was measured after all strains were incubated in PP media for 12 h and 18 h. The results indicated that alkaline phosphatase activity was greater in the ΔLapS strain than in the WT strain and that the complementation strain partially reduced alkaline phosphatase production, whereas compared with the control strain, the LapS^+^ strain significantly reduced alkaline phosphatase activity after incubation for 12 h (Fig. 2), suggesting that the sRNA LapS is associated with alkaline phosphatase production under phosphate-depletion conditions. Additionally, the deletion of LapS significantly increased alkaline phosphatase production, whereas the *lapA* mutation reduced alkaline phosphatase production. These contradictory results indicate that the sRNA LapS can control the transcription or translation of *lapA* under phosphate-depleted conditions.

**Fig 3:**
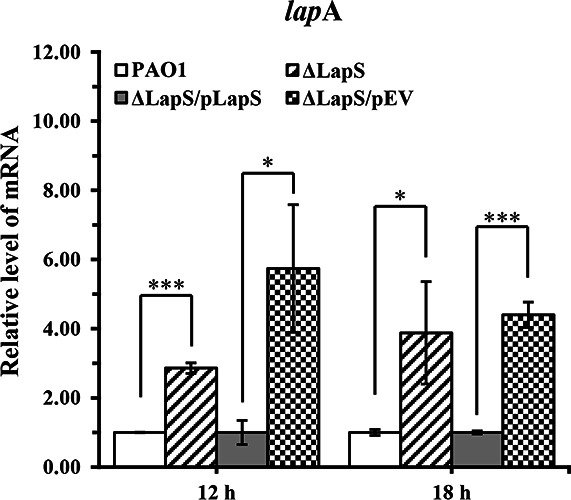
Relative expression levels of *lapA* in *P. aeruginosa* PAO1, ΔLapS, and complementation strains grown under phosphate-depleted conditions. The expression of *lapA* was evaluated using quantitative reverse transcriptase polymerase chain reaction (qRT-PCR). The data are shown as the mean ± standard error of the mean of at least three independent experiments. *, *P* < 0.05; **, *P* < 0.01; ***, *P* < 0.001.

To test this hypothesis, first, the expression of *lapA* was measured via qRT-PCR. The results showed that on average, the expression of *lapA* in the ΔLapS strain was upregulated 2.86-fold compared with that in the WT strain after culture for 12 h and upregulated 3.88-fold after culture for 18 h (Fig. 3). The expression of *lapA* was partially restored in the complementation strain. Compared with that in the control strain, the expression of *lapA* in the LapS^+^ strain was slightly downregulated under phosphate-depleted conditions at 12 h (Fig. S3). Therefore, our data demonstrated that (i) the sRNA LapS inhibited *lapA* expression to reduce alkaline phosphatase production under phosphate-depleted conditions, which is similar to an RNA regulator, and (ii) the LapS mutation did not disturb the *lapA* gene. Moreover, *phoA*, encoding another alkaline phosphatase, is also present in the PAO1 genome (29, 33). Therefore, its expression was also evaluated via qRT-PCR, which revealed that the expression of *phoA* increased by 8.34-fold in the ΔLapS strain when the strain was cultured in PP media for 12 h (Fig. S4A). However, the results did not indicate that LapS could simulate the expression of *phoA* and subsequently regulate alkaline phosphatase production. The alkaline phosphatase activity was markedly reduced when PAO1 was incubated in PP medium for 18 h (Fig. 2), but the transcription of *phoA* increased 5.10-fold compared with that observed after culture for 12 h (Fig. S4B). Collectively, these data indicate that the sRNA LapS affects alkaline phosphatase production by controlling the transcription of *lapA*.

### LapS involvement in the regulation of rhamnolipid production

**Fig 4:**
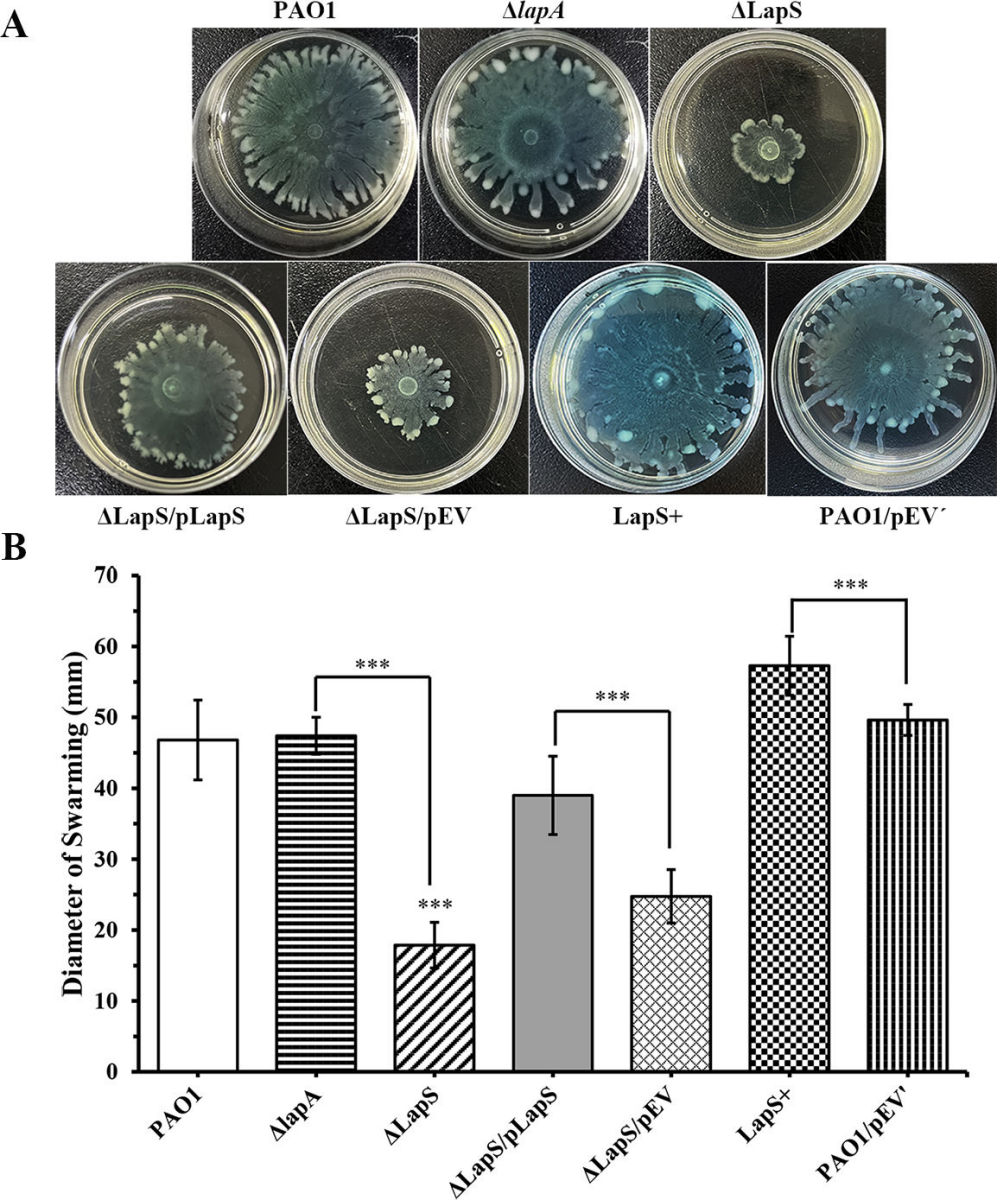
sRNA LapS positively regulates the swarming motility of *P. aeruginosa* PAO1 under phosphate-depleted conditions. One microliter of the culture of the wild-type (WT), ΔLapS, Δ*lapA*, LapS^+^, and LapS complementation strains was spotted onto a swarming medium without phosphate and incubated for 15 h. Swarming motility was evaluated (**A**), and the diameter of the halo was measured (**B**). The data are shown as the mean ± standard error of the mean of at least five independent experiments. *, *P* < 0.05; **, *P* < 0.01; ***, *P* < 0.001.

**Fig 5:**
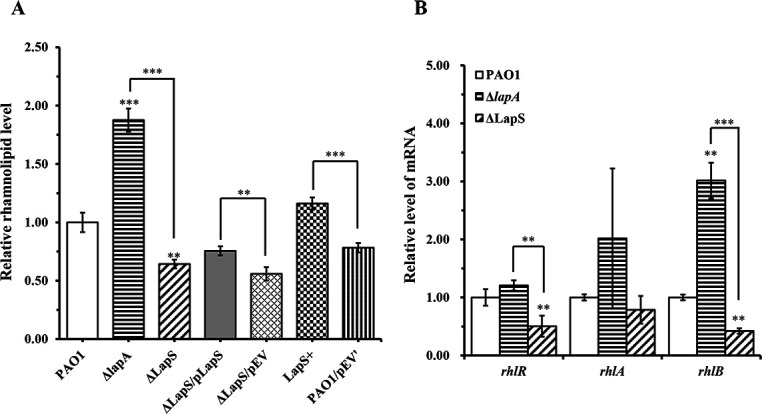
sRNA LapS positively regulates rhamnolipid production by *P. aeruginosa* PAO1 under phosphate-depleted conditions. (**A**) Wild-type (WT), ΔLapS, Δ*lapA*, LapS^+^, and LapS complementation strains were cultured in phosphate-depleted medium for 12 h, after which the amount of rhamnolipid in the supernatants was determined. (**B**) Wild-type (WT), Δ*lapA*, and ΔLapS strains were incubated in phosphate-depleted medium for 12 h, and the expression of *rhlR* and *rhlA*/*B* was evaluated via quantitative reverse transcriptase polymerase chain reaction. The data are shown as the mean ± standard error of the mean of at least three independent experiments. *, *P* < 0.05; **, *P* < 0.01; ***, *P* < 0.001.

Our previous study revealed that the deletion of *lapA* in PAO1 increased rhamnolipid production compared with that in the WT strain under phosphate-depletion conditions (18). In the present study, the deletion of LapS significantly increased the expression of *lapA*. Therefore, we hypothesized that disruption of LapS may decrease rhamnolipid production by increasing *lapA* transcription under phosphate-depleted conditions. To verify this hypothesis, we first investigated rhamnolipid production via a swarming motility assay and found that LapS disruption significantly decreased swarming motility compared with that of the WT and Δ*lapA* strains, whereas the LapS^+^ strain spread throughout the whole plate (60 mm in diameter) after incubation for 15 h, which obviously increased swarming motility (Fig. 4). Swarming motility is used to measure flagellar activity and rhamnolipid production, whereas swimming motility is used to measure flagellar activity (19, 34). Therefore, the swimming motility of these strains was further confirmed via a swimming assay, which revealed that the deletion of LapS did not affect the swimming motility of PAO1 but increased the swimming motility compared with that of the Δ*lapA* strain under phosphate-depleted conditions (Fig. S5). Next, rhamnolipid levels were further evaluated by methylene blue complexation when all the strains were cultured in fresh PP medium for 12 h. The results revealed that LapS deletion indeed reduced rhamnolipid production, whereas LapS overexpression increased rhamnolipid production (Fig. 5A). Finally, the expression of these genes (*rhlR*, *rhlA*, and *rhlB*) related to rhamnolipid production was confirmed via qRT-PCR. The expression of these genes was about 2-fold lower in the ΔLapS strain than in the WT strain and 2-fold to 7-fold lower than that in the Δ*lapA* strain (Fig. 5B). Based on the aforementioned results, LapS indeed regulated rhamnolipid production under phosphate-depleted conditions.

### LapS affects the *las/rhl* system via regulation of the *lapA* level

**Fig 6:**
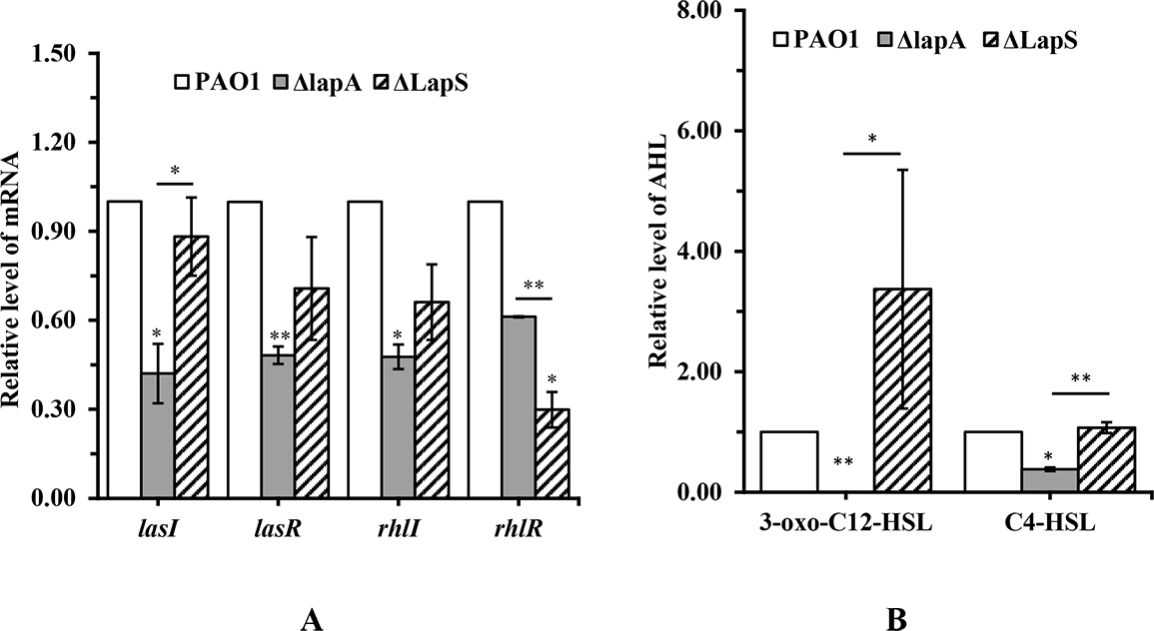
sRNA LapS regulated AHL signals production in *P. aeruginosa* PAO1 via regulation of *lapA* levels under phosphate-depleted stress. (**A**) The WT, Δ*lapA*, and ΔLapS strains were incubated in phosphate-depleted medium for 18 h, and the expression levels of *lasI*/*R* and *rhlI*/*R* were measured using quantitative reverse transcription-polymerase chain reaction. (**B**) WT, Δ*lapA*, and ΔLapS strains were incubated in phosphate-depleted medium for 18 h. C4-HSL and 3-oxo-C12-HSL in the supernatants were extracted and measured using HPLC. The data are shown as the means ± standard errors of the means of at least three independent experiments. *P < 0.05, **P < 0.01, ***P < 0.001.

In our previous study, we showed that *lapA* deletion decreased the levels of AHL signals and subsequently decreased the levels of virulence factors, such as elastase, in *P. aeruginosa* (18). In the present study, LapS deletion induced the transcription of *lapA*. However, the role of LapS in AHL production is unclear. To address this question, first, AHL reporter plate bioassays revealed no significant difference in the violacein halos produced by the ΔLapS and WT strains, whereas the ΔLapS strain produced more violacein halos than did the Δ*lapA* strain when incubated under phosphate-depleted conditions (Fig. S6). These results indicated that the deletion of LapS increased AHL production compared with that of the Δ*lapA* strain. Next, the effects of LapS on the expression of QS-related genes (*lasI*/*R* and *rhlI*/*R*) were measured via qRT-PCR. Compared with the WT strain, LapS deletion interfered with only *rhlR* expression; however, compared with the Δ*lapA* strain, LapS deletion significantly increased the expression level of *lasI* (Fig. 6A). Finally, AHL signals were detected via high-performance liquid chromatography (HPLC), which revealed that LapS deletion increased C4-HSL and 3-oxo-C12-HSL production compared with that of the Δ*lapA* strain under phosphate-depleted conditions (Fig. 6B). Therefore, these data indicate that the sRNA LapS indeed affects AHL signal production via the regulation of *lapA* levels.

### LapS involvement in the biofilm formation of *P. aeruginosa* in a chronic wound model

**Fig 7:**
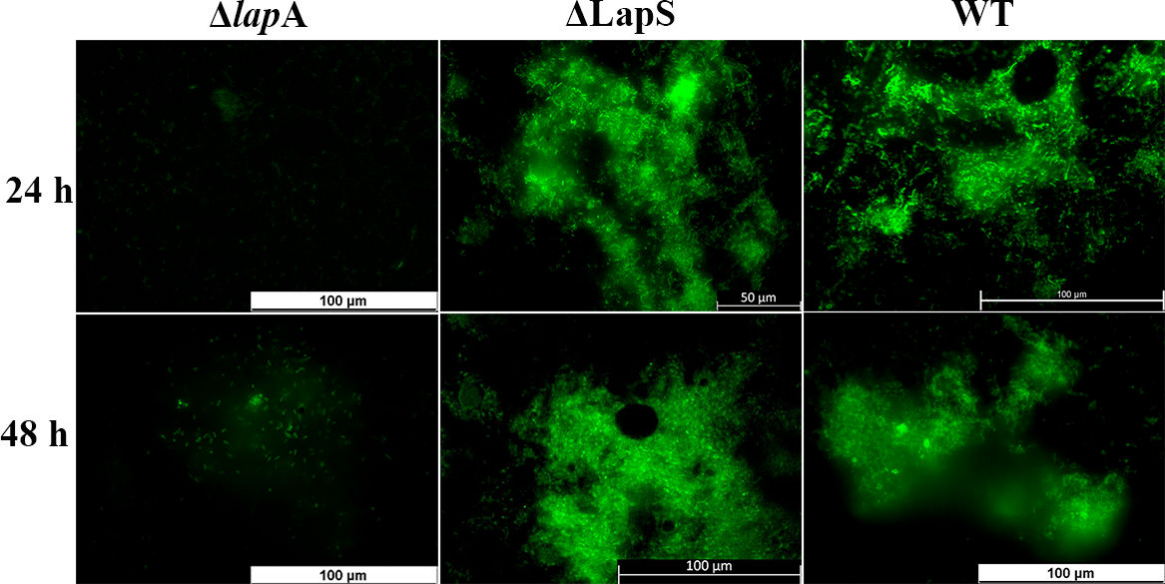
Deletion of LapS induced biofilm formation by *P. aeruginosa* PAO1 in a chronic wound model established with *ex vivo* porcine skin explants.

In our previous study, we reported that the deletion of *lapA* inhibited the biofilm formation of *P. aeruginosa* in porcine skin explants via a decrease in the *las* and *rhl* QS systems and EPS synthesis (18). However, the regulatory role of LapS in *P. aeruginosa* biofilm formation under phosphate-depleted conditions is unknown. Therefore, the ability of the ΔLapS strain to form biofilms was evaluated. Interestingly, compared with the Δ*lapA* strain, LapS deletion induced PAO1 biofilm formation in a chronic wound model established with *ex vivo* porcine skin explants (Fig. 7). Thus, LapS also regulates *P. aeruginosa* biofilm formation in chronic skin wounds. Furthermore, the expression of *lapA* and QS- and EPS-related genes was evaluated via qRT-PCR assays. Although the expression of *lapA* and *las/rhl* systems was upregulated in the biofilms formed by the ΔLapS strain compared with those formed by the WT strain after they were incubated in porcine skin explants for 48 h, there were no significant differences. In contrast, compared with that of the Δ*lapA* strain, the expression of QS-related genes was significantly greater in the biofilm formed by the ΔLapS strain. (Fig. S7). These results indicate that the deletion of LapS induces *P. aeruginosa* biofilm formation in chronic wounds via an increase in *lapA* levels to increase QS activity.

### Disruption of LapS reduces the virulence of *P. aeruginosa* to *C. elegans*

**Fig 8:**
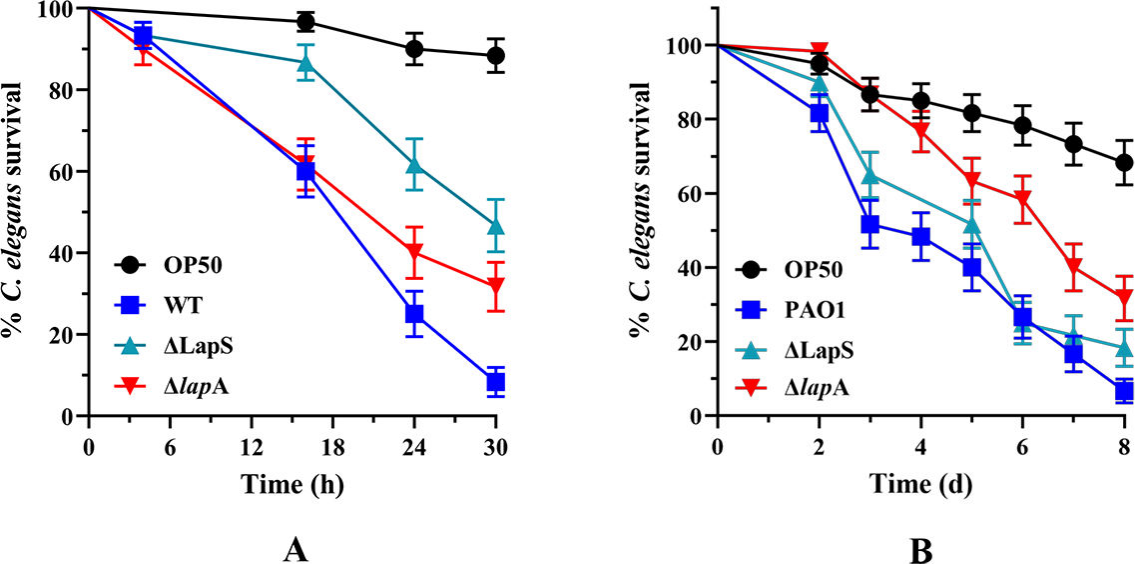
Deletion of LapS decreased the virulence of *P. aeruginosa* PAO1 in a *Caenorhabditis elegans* fast-kill model (**A**), but did not increase the survival ratio of *C. elegans* in a slow-kill assay (**B**). Kaplan–Meier curves of the results are presented and were generated from three independent experiments.

Our results showed that the deletion of LapS decreased the virulence of *P. aeruginosa in vitro* under phosphate-depleted conditions. These findings suggest that the sRNA LapS functions under nonlaboratory conditions, such as during host infection. To verify this possibility, we evaluated the relative pathogenicity of the WT, Δ*lapA*, and ΔLapS strains in a *C. elegans* fast-kill assay. Compared with the WT strain, the ΔLapS strain presented a significant reduction in virulence. The survival rate of *C. elegans* infected with the WT strain for 30 h was only 8%, whereas the survival rate was 47% when infected with the ΔLapS strain for 30 h in a fast-kill assay (Fig. 8A). However, LapS deletion did not affect the survival rate of *C. elegans* in slow-kill assays (Fig. 8B). These results indicate that the sRNA LapS functions to control the virulence of *P. aeruginosa* in nematodes.

### Direct regulation of *putA* by LapS

LapS may affect genes other than *lapA*. To verify this hypothesis, we first used the publicly available database IntaRNA to predict the target genes of LapS (35). The hit with the most extensive base-pairing complementarity was a 16-nucleotide region in the region spanning from –75 to –41 of the *putA* open reading frame that pairs with LapS (Fig. S8A). The product of *putA* is bifunctional proline dehydrogenase, which converts proline to glutamate and is the virulence factor of *P. aeruginosa* in a murine acute pneumonia model (36).

**Fig 9:**
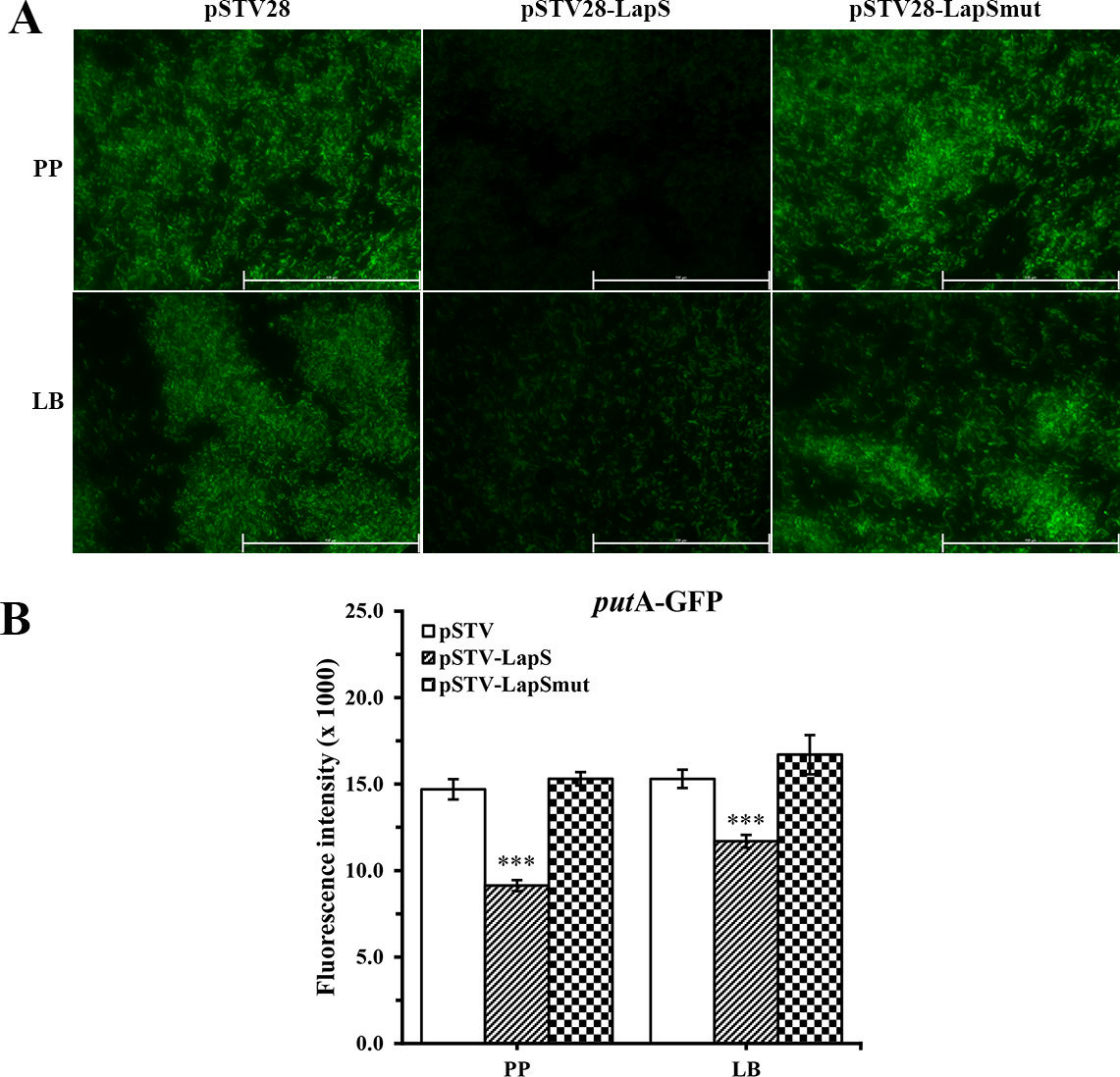
LapS directly targets *putA* and significantly regulates PutA. The plasmids pSTV28, pSTV28-LapS, or pSTV28-LapSmut were co-transformed with a GFP reporter plasmid (pGFPuv-*putA*) containing the sequence of *putA* mRNA in *E. coli* DH5α. Fluorescence was subsequently detected via microscopy (**A**), and the intensity was detected using a microplate reader and expressed in arbitrary units as F485/535 (**B**). The data are shown as the mean ± standard error of the mean of at least three independent experiments. *, *P* < 0.05; **, *P* < 0.01; ***, *P* < 0.001.

To further verify the prediction data for IntaRNA, a green fluorescent protein (GFP) reporter system was constructed (Fig. S8B). The predicted sequences of the LapS-targeted gene were inserted upstream of *gfp* to construct the pGFP-*putA* plasmid. The resulting plasmid was subsequently cotransformed into *E. coli* DH5α with a LapS overexpression vector (pSTV28-LapS) or an empty vector (pSTV28). The fluorescence intensity was measured using a microplate reader and a fluorescence microscope. The intensity of GFP in *E. coli* cells containing pSTV28-LapS was about 61.9% of the intensity in cells containing pSTV28 under phosphate-depletion conditions and 76.5% under phosphate-rich conditions (Fig. 9). To test whether LapS-mediated repression of *putA* occurred through direct pairing via the 16-nucleotide region identified via IntaRNA analysis, the LapS sequence was changed to disrupt base pairing with *putA*. When we used a plasmid (pSTV28-LapSmut) expressing the mutant LapS in place of wild-type LapS, the intensity of GFP was not repressed (Fig. 9). Taken together, these results indicate that LapS directly targets *putA*.

## DISCUSSION

By reanalyzing RNA-seq data from Rockhopper, we identified a number of potential sRNAs related to *P. aeruginosa* biofilms that develop in chronic wounds. Our list included 102 sRNAs, but 29 known sRNAs in *P. aeruginosa* PAO1 were not identified in our RNA-seq analysis. We stress that our analysis did not map the expression of all biofilm-dependent sRNAs. We analyzed only the *P. aeruginosa* PAO1 strain grown in mature biofilms in chronic wound and planktonic states. Furthermore, we used a stringent cutoff for manual curation after prediction by the software. However, our analysis allows for the further discovery of *P. aeruginosa* sRNAs associated with virulence and biofilm formation in wound infections.

RNA-seq technology has been widely used in the transcriptomics analysis of many bacterial studies (37, 38). Furthermore, this technology has also been used to characterize regulatory sRNAs in bacteria (21, 22, 24). The data obtained from our previous RNA-seq experiments allowed us to visualize in detail the changes in the PAO1 transcriptome of biofilms that developed in chronic wound model conditions. The differentially expressed genes were evaluated in a previous study, which revealed that the expression of *lapA*, which encodes an alkaline phosphatase, was highly induced in mature biofilms (28). Therefore, a *lapA* mutant was established, and phenotypic screening experiments were then performed under phosphate-depleted and phosphate-rich conditions. The results suggested that *lapA* is involved in regulating several virulence phenotypes of *P. aeruginosa* under phosphate-depleted conditions, including elastase production, rhamnolipid production, swimming motility, virulence to *C. elegans*, and biofilm formation in chronic wounds (18). In this study, RNA-seq data were reanalyzed, and we found that the novel sRNA LapS, located at the 5´ UTR of *lapA*, was the most differentially expressed sRNA in mature biofilms. However, the following questions remain unanswered. (i) Does LapS control the transcription or translation of *lapA* mRNA? (ii) Does LapS regulate a wide range of virulence phenotypes in *P. aeruginosa* under phosphate-depleted conditions? (iii) Does LapS regulate the biofilm formation of *P. aeruginosa* in chronic wounds? (iv) What is the direct target of LapS other than *lapA*?

To address these questions, the sRNA LapS was further characterized via northern blotting analyses and RACE assays. Indeed, LapS is derived from the 5´ UTR and sequence internal to the *lapA* gene, and it is 197 nucleotides in length, longer than predicted by the software. Importantly, the transcription start sites of *lap*S and *lapA* appear to be the same; two RNA isoforms are produced from this transcription start site: the shorter LapS isoform and the longer *lapS-lapA* isoform. Previous studies have shown that intergenic regions and 3´ UTRs of mRNAs are major sources of regulatory sRNAs, with a few characterized examples of sRNAs derived from 5´ UTRs and/or sequences internal to ORFs (22, 39). Recent studies have indicated that many RNA 3´ ends map upstream of or internal to open reading frames (ORFs) in *E. coli* and *B. burgdorferi* (31, 32). Importantly, Petroni et al. demonstrated that high extracellular spermidine concentrations led to an accumulation of the *potB* 5´ fragment, with a concomitant decrease in the levels of full-length mRNA in *B. burgdorferi*, which is a *potB* 5´ fragment that controls the *potB* mRNA level (31). Morón et al. reported that although the sRNA Rli51 belongs to the 5´ UTR of *mpl* mRNA in *Listeria monocytogenes*, its absence seemed to have a positive effect on *mpl* expression at relatively high levels of transcript induction, which suggests that Rli51 might be a *cis*-acting negative regulatory element of *mpl* transcription (40). However, Thomason et al. demonstrated that the sRNA RhlS derived from the 5´ UTR of *the rhlI* gene stimulated the translation of *rhlI* mRNA to increase C4-HSL production in *P. aeruginosa* (22). Therefore, the regulatory role of sRNAs derived from 5´ UTR and sequences internal to ORFs may differ from that of 5´ UTR-derived sRNAs. In this work, we found that sRNA LapS mutation increased the level of *lapA* mRNA, which indeed differs from the role of the sRNA RhlS in *P. aeruginosa* but is similar to the role of the *potB* 5´ fragment in *B. burgdorferi* and Rli51 in *L. monocytogenes*. To figure out whether the *lapA* gene loses its native transcriptional and translational regulation when LapS is deleted, phenotypic experiments in which *lapA* is regulated were performed. The results demonstrated that the phenotypes of the ΔLapS mutant were the opposite of those of the Δ*lapA* mutant, such as alkaline phosphatase and rhamnolipid production, motility, and biofilm formation. However, LapS overexpression in the *P. aeruginosa* PAO1 strain produced phenotypes opposite to those of the LapS mutant. Therefore, our results suggest that the sRNA LapS indeed controls the level of *lapA* mRNA.

LapS mutation and overexpression were established in *P. aeruginosa* PAO1, and phenotypic screening experiments were performed. We found that LapS is involved in regulating rhamnolipid production and the swarming motility of *P. aeruginosa* under phosphate-depleted conditions. Furthermore, the deletion of LapS reduced the mortality rate of *C. elegans* in the fast-kill assay but did not increase the survival rate of the worm in the slow-kill assay. Fast killing is mediated, at least in part, by low-molecular-weight toxins, including phenazines, and does not require live bacteria, which respond to acute infection; slow killing is due to active infection caused by live *P. aeruginosa* that accumulate in the lumen of the *C. elegans* intestine, develop symptoms of infection, and die over several days, which respond to chronic infection (41, 42). Finally, LapS did not affect the *las/rhl* systems compared with those of the WT strain under phosphate-depleted conditions. Therefore, we concluded that the sRNA LapS is involved in acute infection by *P. aeruginosa*. Additionally, we found that LapS directly targeted *put*A, a virulence factor of *P. aeruginosa* (36). These results reveal the roles of LapS in regulating PAO1 virulence.

Biofilm formation is a common community behavior of *P. aeruginosa* and is regulated by QS (43, 44). Rhamnolipids play crucial roles in the architecture of biofilms (45). Therefore, QS-mediated rhamnolipid production is a positive regulator of biofilm formation. In this study, we found that LapS deletion reduced rhamnolipid production but did not affect AHL signal production, which was consistent with the findings of our previous study (18). Rhamnolipid hyperproduction is unrelated to C4-HSL levels under phosphate-depleted conditions (18). Furthermore, biofilm formation is inversely associated with the motility of *P. aeruginosa* (46). Nevertheless, rhamnolipid synthesis is needed for the swarming motility of *P. aeruginosa* (47). To better investigate the involvement of LapS in biofilm formation and rhamnolipid production, the swarming motility of all strains was investigated under phosphate-depleted conditions. LapS mutation reduced the swarming motility of PAO1, whereas LapS overexpression increased the phenotype, indicating that LapS plays a positive role in rhamnolipid production and swarming motility. Therefore, LapS deletion induced biofilm formation under phosphate-depleted conditions possibly due to reduced swarming motility.

In summary, we revealed that the sRNA LapS negatively regulates the level of *lapA* mRNA, preventing LapA from being overproduced under phosphate-depleted conditions. Therefore, this LapS-*lapA* signaling cascade is beneficial for balancing the virulence regulation of *P. aeruginosa*. Furthermore, LapS interferes with the post-transcriptional regulation of the unlinked gene *putA*, which encodes bifunctional proline dehydrogenase, a virulence factor of *P. aeruginosa*. Collectively, our findings reveal an unstudied regulatory sRNA and highlight the importance of sRNAs in the pathogenesis of *P. aeruginosa* PAO1.

## MATERIALS AND METHODS

### Bacterial strains, plasmids, and growth conditions

The bacterial strains and plasmids used in this study are listed in Table S3. All the strains were cultured in lysogeny broth (10 g of tryptone, 10 g of NaCl, and 5 g of yeast extract per liter) at 37°C under shaking at 150 rpm unless otherwise indicated. Proteose-peptone medium containing 0.4% glucose (PP medium) was used as the phosphate-limiting medium (29, 33). The following antibiotics were used at the indicated concentrations as needed: 100 µg/mL carbenicillin, 50 µg/mL gentamicin, 50 µg/mL nalidixic acid, 50 µg/mL apramycin, and 50 µg/mL chloramphenicol. All antibiotics were purchased from Adamas (Shanghai, China).

### RNA-seq data analysis

The raw RNA-seq data were obtained from our previous study (28). In this study, Rockhopper software was used for alignment and prediction of sRNAs (48). After that, the predicted sRNAs in the >40–<500 nt range were manually retained for further investigation. The expression of sRNAs was calculated via RNA-seq by expectation-maximization (RSEM) (49). Differential expression analysis was performed via the statistical software R package DESeq2 (50) with the following parameters: (i) the Benjamin-Hochberg (BH)-adjusted *P* value (padj) should be less than 10^–3^, (ii) the log_2_FoldChange (log_2_FC) between biofilm and planktonic cells should be greater than 2-fold, and (iii) the normalized count value of each sRNA should be more than 1.00 under either of the conditions. Finally, IntaRNA was used to predict the target genes of sRNAs (35).

### Northern blotting and rapid amplification of cDNA ends (RACE) assays

According to our previous study (18, 28), under phosphate-depleted conditions, *P. aeruginosa* PAO1 can produce more alkaline phosphatase when cultured for 12 h but produces very little alkaline phosphatase at 18 h. However, under phosphate-rich conditions (LB medium), no alkaline phosphatase was produced when PAO1 was cultured for 12 h and 18 h. Therefore, in this work, PAO1 was incubated in PP or LB media for 12 or 18 h, respectively. After incubation, the pellets were collected, and RNA was extracted using a TRIzol Plus RNA Purification Kit (Thermo Fisher, USA) following the manufacturer’s protocols. The genomic DNA was removed using RNase-Free DNase (Qiagen, USA). Finally, each total RNA sample was suspended in 40 µL of RNase-free water, and the quality of the RNA was determined using a Nanodrop 100 spectrophotometer and agarose gel electrophoresis. Only high-quality RNA was used for northern blotting analyses and RACE experiments.

Northern blotting was carried out with the DIG-High Primer DNA Labeling and Detection Starter Kit II (Roche, #11585614910) according to the manufacturer’s protocols. For total RNA, 10 µg was separated on a 1% agarose gel containing 18% formaldehyde at 50 V for 1.5 h. The gels were briefly rinsed in 50 mM NaOH for 15 min, 0.2 M NaOAc (pH 4.6) for 15 min, and 10× SSC for 5 min. The washed RNA was subsequently transferred onto an *N* + nylon membrane (Millipore) overnight and crosslinked by UV crosslinking at 120 mJ/cm^2^ for 1 min. The crosslinked membranes were prehybridized for 2 h at 50°C in DIG Easy Hyb buffer. Oligonucleotide probes were labeled with DIG high prime labeling mix and added to fresh Easy Hyb buffer (40–50 ng/mL), after which the blots were incubated with hybridization buffer overnight at 50°C. After high- and low-stringency washes, the blots were further washed with DIG Wash and Block Buffer (Roche). Anti-DIG antibody (1:10,000 in blocking buffer) was added, and the samples were incubated for 1 h at room temperature and washed in washing buffer. The hybridization signals were visualized using a phosphorimager after the addition of chemiluminescent CSPD for 5 min. The size of each transcript was determined by comparing its corresponding band with that of the RNA marker (Takara, #3586A). 5S rRNA was used as an internal control. The specific probes used in the northern blotting assay are presented in Table S4.

RACE assays were carried out with a GeneRacerTM Kit (Thermo Fisher, #L150001) according to the manufacturer’s protocols. For 5´ RACE of LapS and *lap*A, 1.5 µg of total RNA from 12 h PP culture was treated with calf intestinal phosphatase (CIP) to remove the 5´ cap and expose the 5´ phosphate, thus enabling the ligation of the RNA oligos with T4 RNA ligase. cDNA was generated by reverse transcription with SuperScript III RT and GeneRace OligodT primers. cDNA (0.5 µL) was used for PCR amplification with the 5´ GeneRacer outer primer and the specific primer R1 in a 25 µL total reaction. To avoid nonspecific bands, we reamplified the PCR products with the 5´ GeneRacer inner primer and the specific primer R2. A similar procedure was followed for the 3´ end of LapS characterization, which involved the use of 3´ GeneRacer outer and inner primers and specific primers F. The final PCR products were subsequently cloned and inserted into the pGEM-T vector system (Promega, #A3600) before Sanger sequencing. The specific primers used in the RACE assays are listed in Table S4.

### Construction of LapS-deficient and complementation strains

To avoid interruption of the *lapA* gene, the sRNA LapS was inactivated at lengths ranging from 7 to 107 bp via double-crossover homologous recombination based on the methods of Park et al. with slight modifications (51). Briefly, the pXT02 knockout plasmid based on pKC1139 was constructed by amplifying the ampicillin resistance gene as a selection marker from the pUCP18 plasmid and left- and right-flanking regions of LapS using the genomic DNA of PAO1 as a template. The primer pairs *lapS*-P1-P2, *lapS-*P3-P4, and *ampR*-P1-P2 (Table S4) were designed for the amplification of the left- and right-flaked fragments of LapS and the selection marker. DNA assembly was performed via digestion using restriction enzymes (Thermo Fisher Scientific, USA) and ligation using T4 DNA ligase (New England Biolabs, England) according to the manufacturer’s instructions. The pXT02 plasmid was passaged using *E. coli* S17-1λ-pir and then introduced into the PAO1 strain via conjugation (52). The target region of LapS was disrupted through insertional inactivation via double-crossover homologous recombination. The desired mutant ΔLapS was selected based on its carbenicillin resistance (100 µg/mL) and apramycin-sensitive (50 µg/mL) phenotype, which was verified via PCR with the primer pair LapS-P5-P6 (Table S4) and identified by sequencing. The resulting LapS deletion mutant of PAO1 was termed ΔLapS.

The empty pBBR1MCS-5 vector, a gentamicin-resistant broad-host cloning vector, was used to construct the ΔLapS complementation strain. The whole sequence of LapS was amplified via PCR using the primer pair LapS-P7-P8 (Table S4), for which the genomic DNA of PAO1 was used as the template. The PCR product and the empty vector pBBR1MCS-5 were digested using the restriction enzymes EcoRI and BamHI (Thermo Fisher Scientific, USA), respectively, according to the manufacturer’s protocols. The digested products were then purified and ligated using T4 DNA ligase (New England Biolabs, England) following the manufacturer’s protocols. The ligation products were chemically transformed into *E. coli* DH5α competent cells, and the transformed cells were plated on LB agar plates containing gentamicin. The recombinant plasmid was identified by PCR and then sequenced. Finally, the target plasmid, named pLapS, was chemically transformed into the ΔLapS strain. The ΔLapS strain containing the plasmid pLapS was named the ΔLapS/pLapS strain. The empty pBBR1MCS-5 vector was subsequently transformed into the ΔLapS strain as the control strain, which was named the ΔLapS/pEV strain.

### Construction of the sRNA LapS overexpression strain

The vector pUCP18 was used to develop an overexpression strain for the sRNA LapS in *P. aeruginosa* PAO1 according to a previous study (53). Briefly, the sRNA LapS was amplified from PAO1 genomic DNA using the primer pair *lapS*-P9–P10 by PCR. Following amplification, both the pUCP18 plasmid and the PCR product were digested with EcoRI and HindIII enzymes (Thermo Fisher Scientific, USA) and then ligated using T4 DNA ligase (New England Biolabs, England) according to their protocols. The resulting ligation mixture was then transformed into the *E. coli* DH5α strain and plated on LB agar supplemented with 100 µg/mL carbenicillin. Positive clones were confirmed through PCR and then sequenced. The pUCP18 plasmid containing the sRNA LapS and the empty pUCP18 vector were separately introduced into the component cells of WT PAO1 to obtain the LapS^+^ and PAO1/pEV´ strains, respectively.

### Alkaline phosphatase activity assays

A single colony from the LB plates was inoculated into 2 mL of LB medium and incubated at 37°C overnight. Furthermore, 5 µL of each overnight culture was added to 5 mL of fresh PP medium and then incubated at 37°C under shaking at 150 rpm for 12 h and 18 h, respectively. Then, 200 µL of each culture was taken, and the absorbance was detected at OD600 via a microplate reader (TECAN Spark, Switzerland). Furthermore, 1 mL of each culture was removed and centrifuged for 5 min at 10,000 rpm. The supernatants were collected and passed through a 0.22 µm syringe filter, and the alkaline phosphatase activity in the filtrate was evaluated via an alkaline phosphatase assay kit (Beyotime, Beijing, China) according to the manufacturer’s protocols. The results were normalized to the OD600. Each experiment was performed at least in triplicate.

### Motility assays

Motility assays were performed via a previously described method (54). Media for swimming (PP medium containing 0.3% agar) and swarming (PP medium containing 0.4% agar) assays were prepared. One microliter of overnight culture was then spotted onto the center of a plate (60 mm diameter) containing each type of medium. The plates were incubated at 37°C for 24 h, after which the diameter of the motility zone developed by each strain was measured. Each experiment was performed at least in triplicate.

### Rhamnolipid assay

Rhamnolipid production was measured by methylene blue complexation via the methods of Pinzon et al. with slight modifications (55). Briefly, 5 µL of each overnight culture was added to 5 mL of fresh PP medium and incubated at 37°C under shaking at 150 rpm for 12 h. The supernatant (1 mL) was acidified with 1 M HCl, and the rhamnolipid was extracted with 5 mL of chloroform. Then, 3 mL of the chloroform extract was added to a new tube and allowed to react with 100 µL of methylene blue (1 g/L) and 5 mL of distilled water. Finally, 200 µL of the chloroform layer was collected, and the OD638 of each sample was measured using a microplate reader (TECAN Spark, Switzerland). Each experiment was performed at least in triplicate.

### AHL detection assays

*Chromobacterium violaceum* CV026 is used as a biosensor to visualize AHLs with N-acylside chains from C4–C8 in length produced by gram-negative bacteria (21, 56). In this study, one colony of CV026 was inoculated into 2 mL of LB medium and incubated at 28°C overnight. The CV026 culture was added to warm PP agar (1.5%) medium at a ratio of 1:100, and the mixture was then poured immediately over the surface of PP agar plates prepared in Petri dishes. When the agar solidified, five wells (5 mm diameter for each well) were prepared in each plate, whose bottoms were sealed with warm agar solution. Next, 25 µL of each overnight culture was added to each well. Moreover, 25 µL of LB was prepared under the same conditions as those used for the control. Violacein halo production was observed after incubation at 28°C for 48 h, and the diameter of each violacein halo was measured.

AHL signaling molecules produced by *P. aeruginosa* were extracted and measured via a method described previously by our group, with slight modifications (54). Briefly, AHL production was determined by inoculating 200 µL of overnight culture into 200 mL of PP medium. After 18 h of cultivation at 37°C, the sterile supernatant was collected and extracted with acidified ethyl acetate on the basis of a previously described method (57). AHLs produced by bacteria were analyzed via ultraviolet absorbance at 210 nm via an HPLC system (Shimadzu, Japan) equipped with a C18 column. Mobile phase A was water, whereas mobile phase B was methanol. The flow rate was set as 0.8 mL/min. The injection volume was 20 µL. The peaks corresponding to C4-HSL and 3-oxo-C12-HSL were identified based on the retention times of commercial C4-HSL and 3-oxo-C12-HSL standards, respectively (Aladdin, Shanghai, China), using the same HPLC protocol. Each experiment was performed at least in triplicate.

### Biofilm formation assays

An *ex vivo* biofilm formation assay was performed in porcine skin explants on the basis of a method previously described by our group (28). Briefly, 10 µL (106 colony-forming units (CFUs)) of overnight culture mixture was added to each explant well. Soft agar plates were statically incubated at 37°C. All explants were transferred to fresh soft agar plates (containing only 0.5% agar) containing irgasan (25 µg/mL) each day. Then, 10 µL of LB medium was added to each explant well, and the plate was incubated under the same conditions as those used for the negative control. To measure the biofilms that developed in the porcine skin explant wells, the explants were gently washed with 10 mL of sterile phosphate-buffered saline (PBS) three times to remove loosely bound cells. The explants were then sonicated in 2 mL tubes containing 1 mL of sterile PBS for 30 s, followed by vigorous mixing. Proper dilutions were made with sterile PBS, and the samples were plated on *Pseudomonas* isolation agar plates. The plates were incubated at 37°C overnight, after which the bacterial colonies were counted. One set of washed explants was stained using an acridine orange/ethidium bromide staining kit (Sangon Biotech, China) according to the manufacturer’s instructions. Imaging of biofilms in explant wells was performed under a fluorescence microscope (Leica Microsystems, Germany). The imaging areas were selected at the center of the reservoir to avoid edge effects. Each experiment was performed at least in triplicate.

### qRT-PCR assay

Total RNA was extracted using the Spin Column Bacterial Total RNA Purification Kit (Sangon Biotech, China) according to the manufacturer’s protocols. Complementary DNA was synthesized using MonScript RTIII Super Mix with a dsDNase Kit (Monad, China) following the manufacturer’s protocols. The primers used for this assay were designed using Primer3 software, and the sequences are listed in Table S4. The qRT-PCR assay was performed in a 20 µL reaction volume using MonAmp SYBR Green qPCR Mix (Monad, China) following the manufacturer’s instructions. These reactions were performed using a LightCycler 96 instrument (Roche Diagnostics, USA) with the following cycle parameters: 95°C for 30 s, 40 cycles of 95°C for 5 s, 60°C for 30 s, and 95°C for 15 s. All the experiments were performed in triplicate, and the data were recorded. The results were normalized to those of the housekeeping genes *rpsL* for *in vitro* studies (RNA-seq, data not shown) and *recA* for biofilms according to our previous studies (28), which were used as internal reference genes for the planktonic state and biofilm, respectively. Fold changes (FCs) between the experimental group and the control group were calculated via the 2^−∆∆Ct^ method.

### *C. elegans* fast-kill and slow-kill assays

*C. elegans* killing assays were performed using WT N2 worms for each condition using methods previously described by our group (58). For the fast-kill assay, 20 worms at the L4 stage were transferred to lawns of the WT, Δ*lapA*, and ΔLapS strains on PGS agar plates (1% peptone, 1% NaCl, 1% glucose, 0.15 M sorbitol, and 1.7% agar), respectively. All the experimental plates were supplemented with nalidixic acid (5 µg/mL) to inhibit the growth of OP50 and 5-fluoro-2′-deoxyuridine (FUDR, 25 µg/mL; Adamas, China) to inhibit egg production. The plates were incubated at 25°C, and nematodes were scored for survival at 4, 16, 24, and 30 h. Furthermore, 20 worms at the L4 stage were added to lawns of *E. coli* OP50 on the same plates without nalidixic acid and incubated under the same conditions as those used for the reference treatment.

For the slow-kill assay, 20 worms at the L4 stage were moved to lawns of the WT, Δ*lapA,* and ΔLapS strains on SK assay plates (0.35% peptone, 0.3% NaCl, 5 µg/mL cholesterol, 2% agar, 1 mM CaCl_2_, 1 mmol/L MgSO_4_, or 25 mmol/L KH_2_PO_4_). All the experimental plates were supplemented with nalidixic acid (5 µg/mL) to inhibit the growth of OP50 and FUDR (25 µg/mL) to inhibit egg production. The plates were incubated at 25°C, and nematodes were scored for survival every 24 h for 8 days. At the same time, 20 worms were added to lawns of *E. coli* OP50 on the same plates without nalidixic acid and incubated under the same conditions as those used for the reference. Each experiment was performed at least in triplicate.

### Fluorescent reporter assays

To investigate the effect of LapS on the translation of *putA*, the GFP reporter plasmid pGFPuv and the sRNA overexpression plasmid pSTV28 were used in this study according to previous studies (21). Additionally, both vectors can simultaneously survive in *E. coli* cells. The sequence of *put*A containing putative binding sites for LapS was amplified via PCR from PAO1 genomic DNA using the cloning primer pair *putA*-P1-P2, as described in Table S4. The pGFPuv plasmid and PCR product were subsequently digested with HindIII/XbaI enzymes and then ligated via T4 DNA ligase according to their protocols. The resulting ligation mixture was transformed into *E. coli* DH5α and plated on LB agar supplemented with 100 µg/mL carbenicillin. Positive clones were confirmed through PCR and sequenced. The sequence of LapS was subsequently amplified via PCR from PAO1 genomic DNA using the cloning primer pair LapS-P9-P10, as described in Table S4. Both the pSTV28 plasmid and LapS PCR product were digested with EcoRI/BamHI enzymes and then ligated using T4 DNA ligase according to their protocols. The ligation mixture was transformed into *E. coli* DH5α strain containing pGFP-*putA* and plated on LB agar supplemented with 50 µg/mL chloramphenicol and 100 µg/mL carbenicillin. Positive clones were confirmed through PCR and sequenced. Moreover, the empty pSTV28 vector was introduced into *the E. coli* DH5α strain containing pGFP-*putA* as control. Furthermore, to further investigate the complementary sites between LapS and *putA*, pSTV28-LapS_mut_, which carried a mutated sequence in the complementary site for *putA*, was generated using fusion PCR. The sequence chosen for mutation was CGCCGCC, which was mutated to GAGGAGG. The plasmid pSTV28-LapS_mut_ was subsequently transformed into *E. coli* DH5α containing the pGFP-*putA* plasmid.

After cotransformation, the cultures were incubated in PP or LB media at 37°C and 150 rpm for 18 h. The pellets were collected via centrifugation, washed, and resuspended in 0.9% NaCl. Then, 200 µL of culture was transferred to a black polystyrene 96-well plate. The fluorescence intensity (F485/535) was measured using a microplate reader (TECAN Spark, Switzerland). Finally, 10 µL of each suspension was added to a slide, after which the fluorescence intensity was observed using a fluorescence microscope (Leica Microsystems, Germany). Each experiment was performed at least in triplicate.

### Statistical analysis

All the experiments were performed at least in triplicate and were repeated on different days unless otherwise stated. The results are presented as the means ± standard deviations. One-way analysis of variance was performed along with Student’s *t*-test to determine statistically significant differences. A *P* value of <0.05 was considered to indicate statistical significance. The log-rank Mantel-Cox test was performed using Prism GraphPad (version 9) software (San Diego, CA, USA) to compare the nematode survival rates between the experimental and control groups.

## Data Availability

All the RNA-seq data in this study are available in the NCBI database under BioProject no. PRJNA688537. All the data generated or analyzed during this study are included in the article and its supplemental material files.

## SUPPLEMENTAL MATERIAL

Supplemental figures spectrum.01303-24-s0001.pdf Figures S1 to S8.

Table S1 spectrum.01303-24-s0002.xlsx sRNAs were predicted with Rockhopper after manual curation.

Table S2 spectrum.01303-24-s0003.xlsx Differentially expressed sRNAs in biofilms compared with planktonic state.

Table S3 spectrum.01303-24-s0004.docx Bacterial strains and plasmids used in the present study.

Table S4 spectrum.01303-24-s0005.docx Sequence of the primers used in this study.

ASM does not own the copyrights to Supplemental Material that may be linked to, or accessed through, an article. The authors have granted ASM a non-exclusive, world-wide license to publish the Supplemental Material files. Please contact the corresponding author directly for reuse.
