# Supplementary material for: Regulatory roles of an sRNA derived from the 5´ UTR and sequence internal to lapA in Pseudomonas aeruginosa PAO1
Source: Microbiol Spectr. 2025 Apr 22;13(6):e01303-24. doi: 10.1128/spectrum.01303-24 (PMC12131859; doi:10.1128/spectrum.01303-24)
Supplement: Supplemental figures — Figures S1 to S8. [file spectrum.01303-24-s0001.pdf]

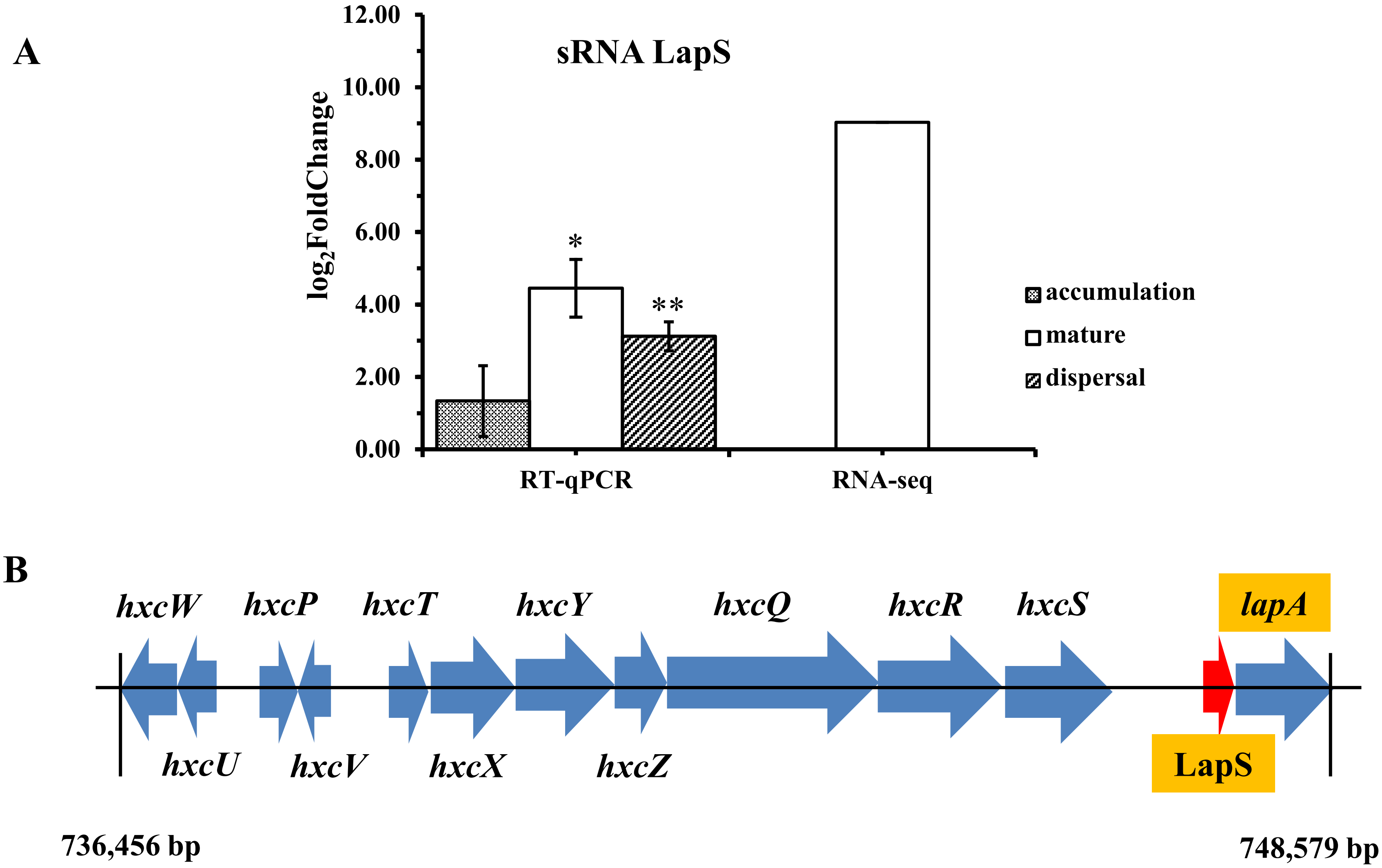

FIG S1 The relative expression of the sRNA LapS in *Pseudomonas aeruginosa* PAO1 biofilms developed from porcine skin explants (A) and the location of LapS in the PAO1 genome (B). The data indicate the $\log_2$FC expression of genes in biofilm cells compared to planktonic cells. The data are shown as the mean ± standard error of the mean of at least three independent experiments. *, $p < 0.05$; **, $p < 0.01$; ***, $p < 0.001$.

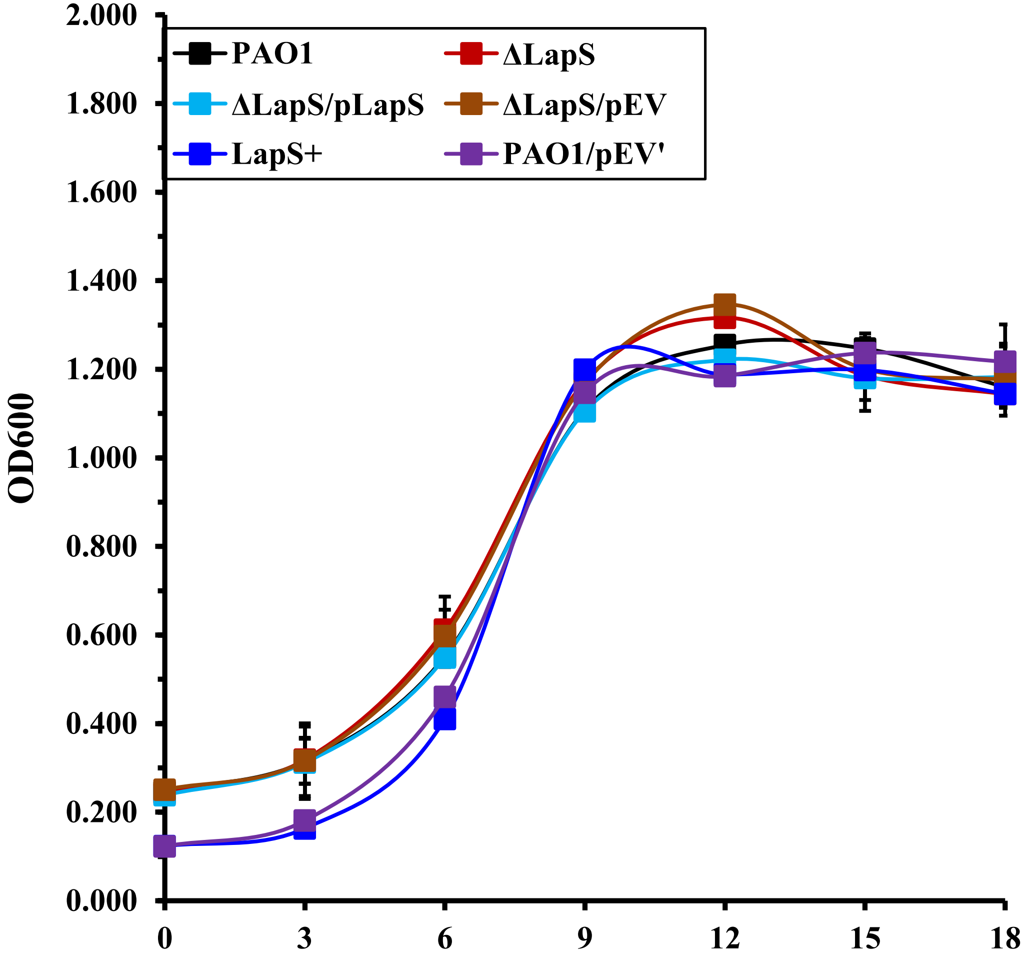

FIG S2 The growth curves of the *Pseudomonas aeruginosa* PAO1, ΔLapS, $LapS^+$, and complementation strains incubating phosphate-depleted medium.

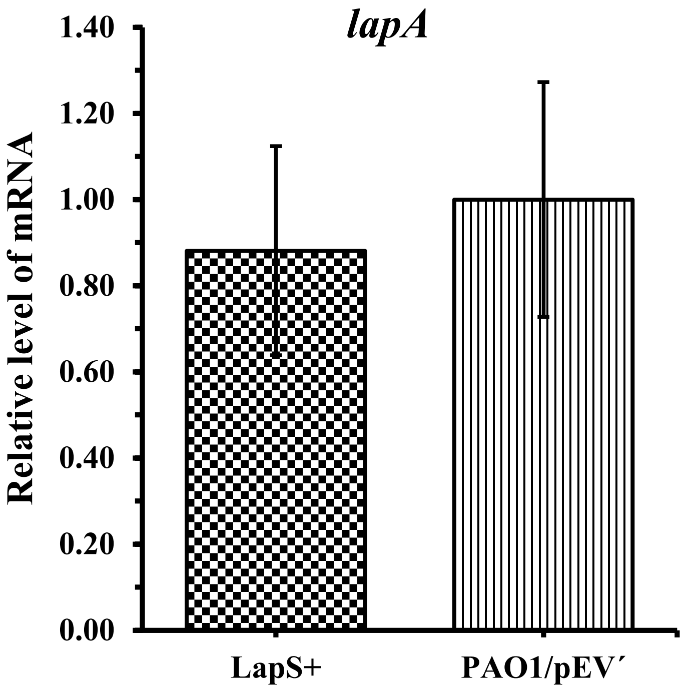

FIG S3 The effects of sRNA LapS on *lapA* transcription under phosphate depletion conditions. Relative expression levels of *lapA* in LapS overexpression strain ($LapS^+$) and its control strain (PAO1/pEV´) was evaluated using quantitative reverse transcriptase polymerase chain reaction (qRT–PCR), when they were incubated at 37^oC for 12 h in phosphate-depleted medium. The data are shown as the mean ± standard error of the mean of at least three independent experiments. *, $p < 0.05$; **, $p < 0.01$; ***, $p < 0.001$.

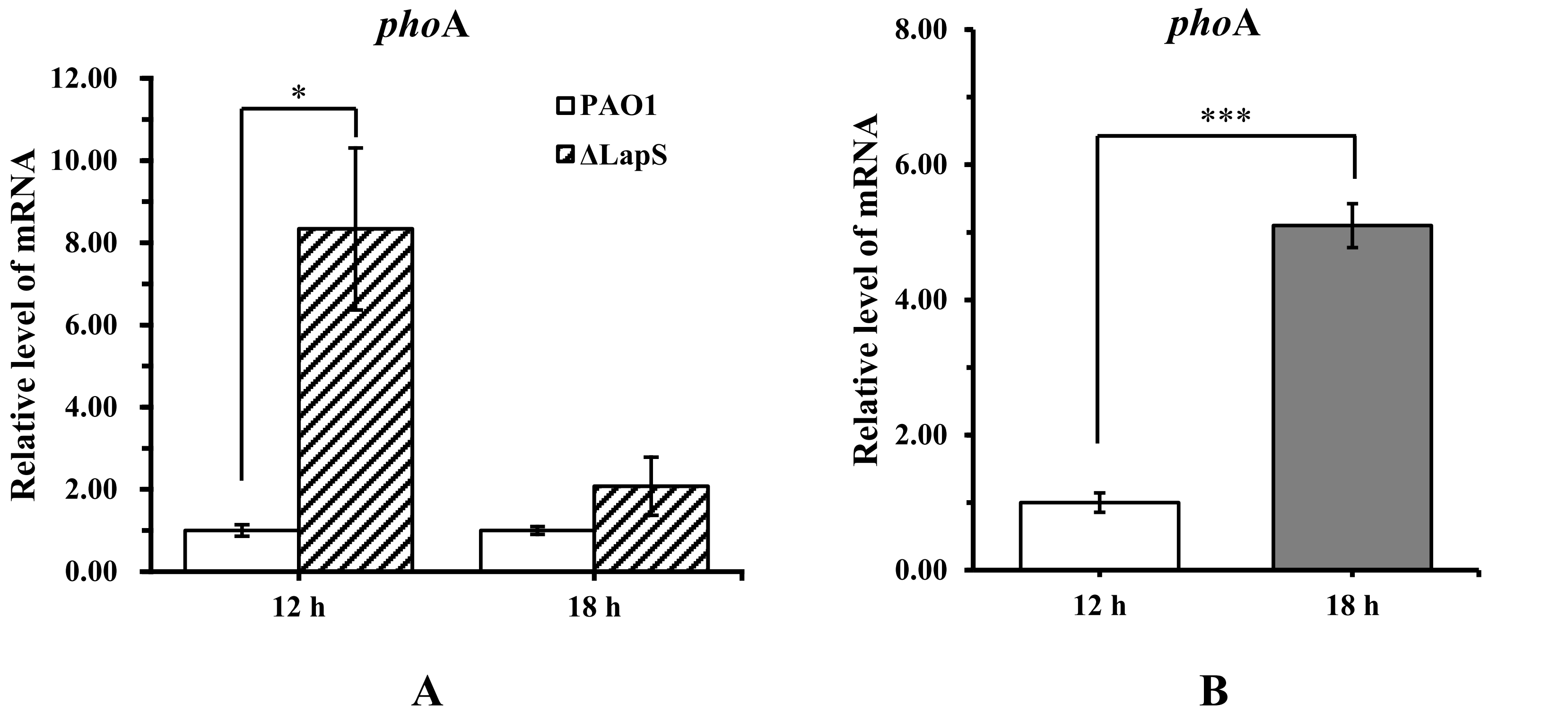

FIG S4 Relative expression levels of *pho*A gene in ΔLapS and wild-type (WT) strains when grown at phosphate-depleted stress for 12 h and 18 h, respectively. (A) Relative expression levels of *pho*A gene in ΔLapS strain compared with WT strain. (B) Relative expression levels of *pho*A gene when WT strain was culture for 12 h and 18 h. The expression of *pho*A was measured using quantitative reverse transcriptase polymerase chain reaction. Data are shown as mean ±standard error of the mean of at least three independent experiments. *, $p<0.05$; **, $p<0.01$; ***, $p<0.001$.

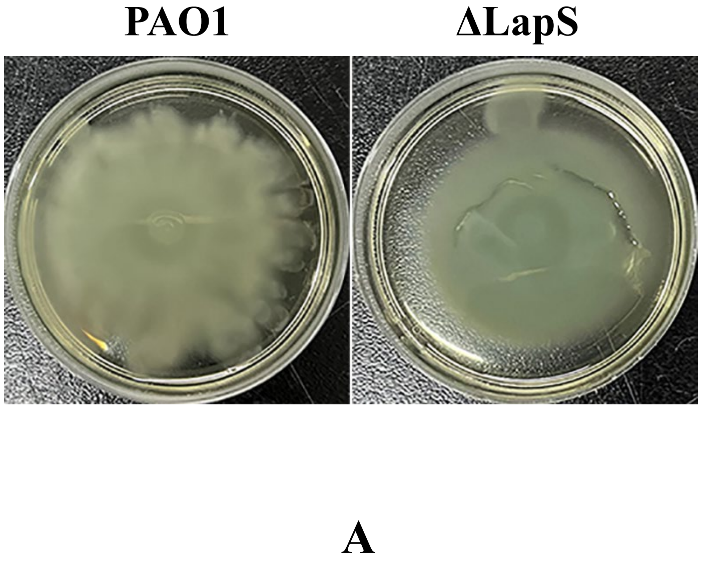

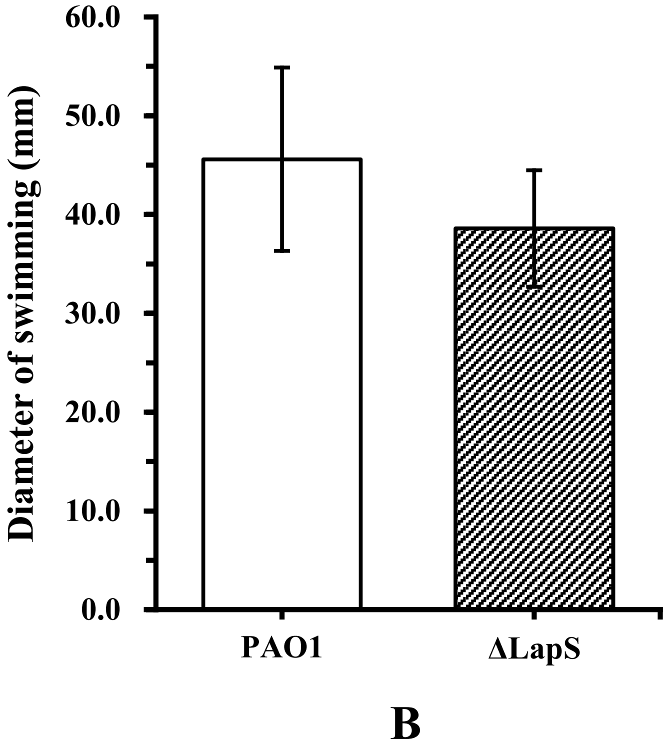

FIG S5 Deletion of LapS did not effect on swimming motility of *Pseudomonas aeruginosa* PAO1 at phosphate-depleted stress. One μL of culture of Wild-type (WT) and ΔLapS strains was spotted onto the swimming medium without phosphate and incubated for 24 h. Swimming motility was evaluated (A), and the diameter of the halo was measured (B). Data are shown as mean ± standard error of the mean of at least five independent experiments. *, $p<0.05$; **, $p<0.01$; ***, $p<0.001$.

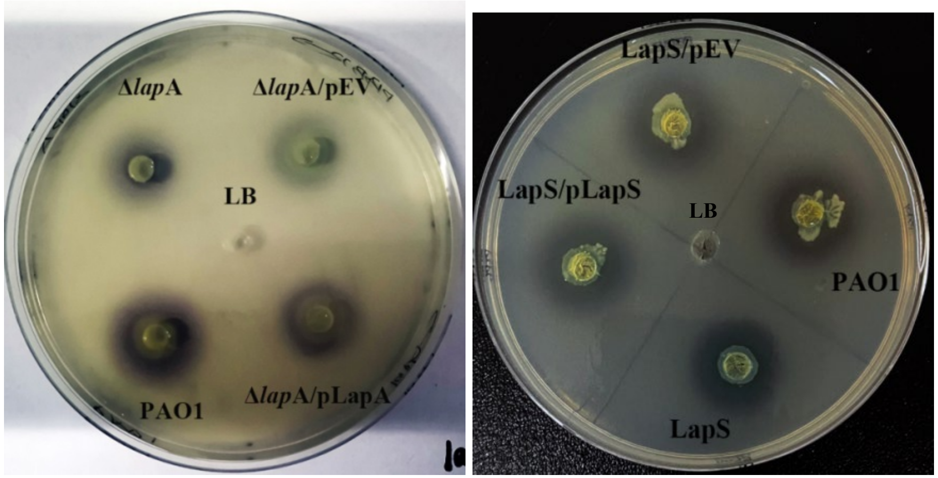

A **B**

FIG S6 AHLs produced by WT, Δ*lapA*, and ΔLapS strains was detected with *C. violaceum* CV026 under phosphate-depleted conditions. Each strain was added to the wells of phosphate-depleted agar plates *containing C. violaceum* CV026 and incubated at 28°C for 48 h; the violacein halo production was evaluated. (A) AHLs produced by WT, Δ*lapA*, and complementary strains [18]; (B)) AHLs produced by WT, ΔLapS, and complementary strains.

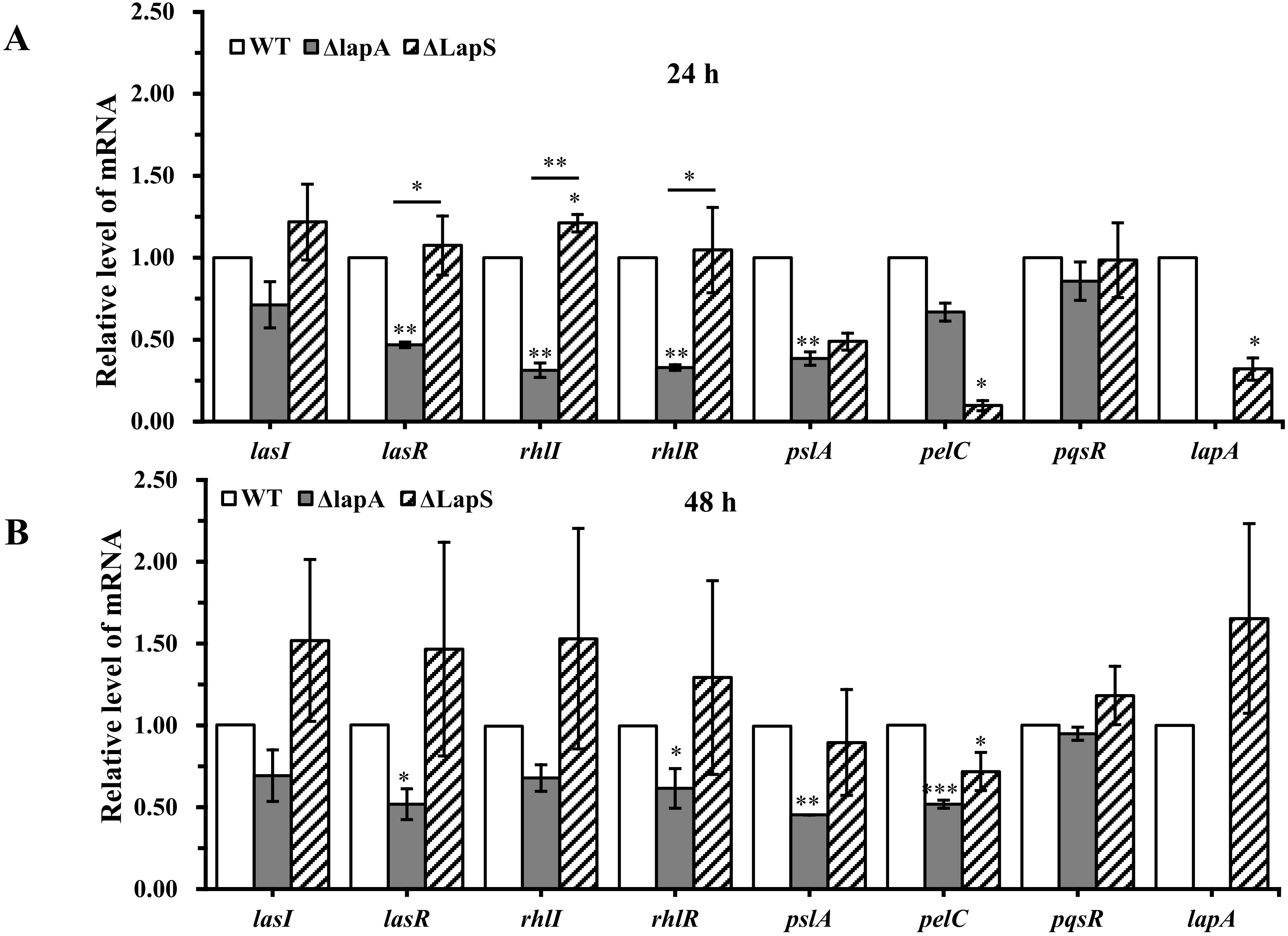

FIG S7 The sRNA LapS showed significant regulations in the *las* and *rhl* systems of *Pseudomonas aeruginosa* PAO1 in porcine skin explants. Wild-type (WT), Δ*lapA*, and ΔLapS strains were cultured in wells containing porcine skin explants and incubated for 24 h and 48 h, and the expression levels of genes related to quorum sensing systems and EPS production in *Pseudomonas aeruginosa* were measured using quantitative reverse transcriptase polymerase chain reaction. (A) Relative expression levels of these genes when the WT, Δ*lapA*, and ΔLapS strains were incubated for 24 h. (B) Relative expression levels of these genes when the WT, Δ*lapA*, and ΔLapS strains were incubated for 48 h. Data are shown as mean ±standard error of the mean of at least three independent experiments. *, $p<0.05$; **, $p<0.01$; ***, $p<0.001$.

```
Target (top)    : putA (PA0782)
Query  (bottom) : LapS

      -75                        -41
        |                         |
      5'-       C     AA  GACG       ACUUC..AGC-3'
        GGCGGCG  GUGC   CCGGGC  UG    CCUGGCCGG
        |||||||  ||||   ||||||  ||    ||||||||
        CCGCCGC  CACG   GGCCCG  AC    GGAUCGGCC
3'-CUU..GUACU     GG   AAC    AA  GUAG      AGCUUCCC-5'
        |                         |
       47                         9

Energy                        : -24.58 kcal/mol
Hybridization Energy          : -55.34 kcal/mol
Unfolding Energy - Target     : 16.02 kcal/mol
Unfolding Energy - Query      : 14.74 kcal/mol
Position - Target RNA         : -75 -- -41
Position - Query RNA          : 9 -- 47
Position Seed - Target RNA    : -75 -- -69
Position Seed - Query RNA     : 41 -- 47
```

A

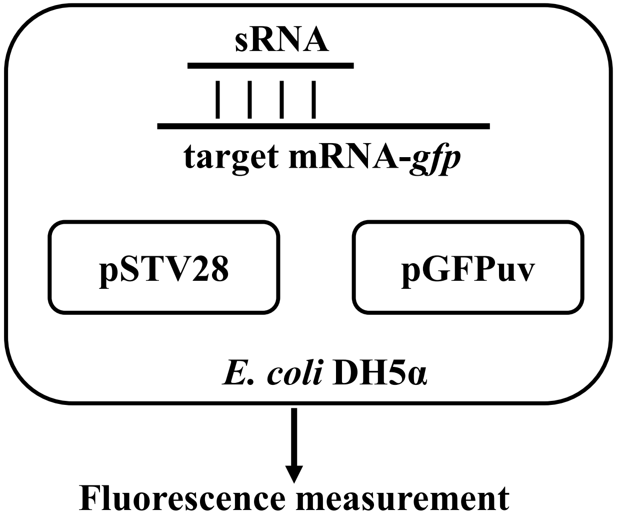

B

FIG S8 LapS directly targeted *putA* at phosphate-depleted stress. (A) LapS and its putative binding sequence in the *putA* mRNA. (B) A green fluorescent protein (GFP) reporter system was constructed to investigate the direct interactions between LapS and its target *putA*.
