# Supplementary material for: Regulatory roles of an sRNA derived from the 5´ UTR and sequence internal to lapA in Pseudomonas aeruginosa PAO1
Source: Microbiol Spectr. 2025 Apr 22;13(6):e01303-24. doi: 10.1128/spectrum.01303-24 (PMC12131859; doi:10.1128/spectrum.01303-24)
Supplement: Table S3 — Bacterial strains and plasmids used in the present study. [file spectrum.01303-24-s0004.docx]

**Table S3** Bacterial strains and plasmids used in the present study.

| **Strains and plasmids** | **Genotype or phenotype** | **Source or reference** |
| --- | --- | --- |
| **strains** | | |
| ***E. coli*** |  |  |
| DH5α | F^–^,φ80,lacZΔM15,Δ(lacZYA-argF)U169,  endA1,recA1,hsdR17(r_k_^–^,m_k_^+^) supE44, λ^–^,thi-1,gyrA96,relA, phoA | Our lab |
| S17-1λ-*pir* | RP4-2(Km::Tn7,Tc::Mu-1), pro-82, LAMpir, recA1, endA1, thiE1, hsdR17, creC510 | Our lab |
| OP50 | Uracil auxotroph, useful for growing *C. elegans* | Our lab |
| ***Pseudomonas aeruginosa*** | | |
| PAO1 | Wild type | Our lab |
| Δ*lap*A | *lap*A deletion strain | Our lab |
| ΔLapS | LapS deletion strain | This study |
| ΔLapS/pLapS | ΔLapS complementation strain | This study |
| ΔLapS/pEV | ΔLapS strain containing plasmid pBBR1MCS-5 | This study |
| LapS^+^ | WT strain containing plasmid pUCP18-LapS to product LapS overexpression strain | This study |
| PAO1/pEV´ | WT strain containing empty pUCP18 vector | This study |
| **Plasmids and vectors** | | |
| pKC1139 | A suicide vector system using the homologous recombination, Apr^R^ | Our lab |
| pXT02 | Homologous recombination plasmid based on pKC1139, Tc^R^, Apr^R^ | This study |
| pBBR1MCS-5 | Gm^R^; broad-host-range vector, P_lac_ | Our lab |
| pLapS | Gm^R^; HidIII/XbaI fragment containing LapS in pBBR1MCS-5 | This study |
| pUCP18 | Amp^R^; used for template for amplifying ampicillin resistance gene, LapS overexpression in PAO1 strain | Our lab |
| pGFP_uv_ | Amp^R^; GFP reporter plasmid | Our lab |
| pGFP-*putA* | Gm^R^; HidIII/XbaI fragment containing *putA* in pGFPuv | This study |
| pSTV28 | Cm^R^; sRNA overexpression plasmid | Our lab |
| pSTV28-LapS | Cm^R^; EcoRI/BamHI fragment containing LapS in pSTV28 | This study |
| pSTV28-LapS_mut_ | Cm^R^; EcoRI/BamHI fragment containing LapS mutant in pSTV28 | This study |
