# Supplementary material for: Regulatory roles of an sRNA derived from the 5´ UTR and sequence internal to lapA in Pseudomonas aeruginosa PAO1
Source: Microbiol Spectr. 2025 Apr 22;13(6):e01303-24. doi: 10.1128/spectrum.01303-24 (PMC12131859; doi:10.1128/spectrum.01303-24)
Supplement: Table S4 — Sequence of the primers used in this study. [file spectrum.01303-24-s0005.docx]

**Table S4** Sequence of the primers used in this study.

| **Primer** | **Sequence (5’ to 3’)** |
| --- | --- |
| LapS-F | AGCGGCCCCTTCGACCGG |
| LapS-R | GCATCCCTAGCCGGTCGAA |
| r*lapA*-R1 | GCCGGTCACGGCATAGCTGAAGTT |
| r*lapA*-R2 | CACGCTCAGCGAGGCAGCGATCA |
| r*lapS*-F1 | CCCTTCGACCGGCTAGGGATGCAA |
| r*lapS*-F2 | CTCAACGTACCAACACCACGATGAA |
| LapS_P1 | CCCAAGCTT(HindIII)TCACCGCGTTCATCTACCCG |
| LapS_P2 | TGCTCTAGA(XbaI)GCCGCTGGGGATAAAACTCG |
| LapS_P3 | CGCGGATCC(BamHI)CATGTTCAAGCGCTCTCTG |
| LapS_P4 | CCGGAATTC(EcoRI)TCACGATGGCGCTCTTCCAG |
| LapS_P5 | TGCGAACACCCGAGGAAAG |
| LapS_P6 | GGCATAGCTGAAGTTGGCCG |
| LapS_P7 | CCGGAATTC(EcoRI)TGCGAACACCCGAGGAAAG |
| LapS_P8 | CGCGGATCC(BamHI)CATGGCTTGGGCGGATACGA |
| LapS_P9 | CCGGAATTC(EcoRI)CCCTTCGACCGGCTAGGGATG |
| LapS_P10 | CGCGGATCC(BamHI)GAAGAATCTCCTTTCATCGTGG |
| LapS_P11 | GTCGTCTTGCATGACCTCCTCCCGTGCTTGCCGGGCTTTG |
| LapS_P12 | CCGGCAAGCACGGGAGGAGGTCATGCAAGACGACTTCCTC |
| *ampR*_P1 | TGCTCTAGA(XbaI)CGCGGAACCCCTATTTGT |
| *ampR*_P2 | CGCGGATCC(BamHI)GGTCTGACAGTTACCAATGCT |
| *putA*_P1 | CCCAAGCTT(HindIII)GCCGCGCCGCCGGCACAA |
| *putA*_P2 | TGCTCTAGA(XbaI)CTCGGCGTCGGCCAGCGA |
| *lasI*_P2 | ATTCGCCAGCAACCGAAAAC |
| *lasR*_P1 | TCGAACATCCGGTCAGCAAA |
| *lasR*_P2 | GTTCACATTGGCTTCCGAGC |
| *rhlA*_P1 | TTTCAACGTGGTGCTGTTCG |
| *rhlA*_P2 | GATCAGCGCCAGGAGGATTT |
| *rhlB*_P1 | GGATGATCGAGCCGGTCTAC |
| *rhlB*_P2 | TACTTCTCGTGAGCGATGCG |
| *rhlC*_P1 | TGTTCAATCCTGGCGACGAC |
| *rhlC*_P2 | GATCGCTGTGCGGTGAGTT |
| *rhlI*_P1 | CATCCGCAAACCCGCTACAT |
| *rhlI*_P2 | GGGTTTCGCTGCACAGGTA |
| *rhlR*_P1 | TGAGGAATGACGGAGGCTTT |
| *rhlR*_P2 | AGGCGTAGTAATCGAAGCCC |
| *plsA*_P1 | TGATCTTCTGGTTCACCGGC |
| *plsA*_P2 | GGTACATGCCGCGTTTCATC |
| *pelC*_P1 | TCCAGCTTCACCAGCGAAAG |
| *pelC*_P2 | GCGCCTGGGAATAATTGAGC |
| *psqR*_P1 | ATAGCCTGGCGACGATCAAG |
| *psqR*_P2 | CACTGGTTGAAGCGGGAGAT |
| sRNA0078_P1 | ACCGGCTAGGGATGCAAAG |
| sRNA0078_P2 | ATCGTGGTGTTGGTACGTTGA |
| *lapA*_P1 | GTTCACTATGCCGGTAGCGA |
| *lapA*_P2 | CCGTAGGTGCCGTTGTAGTT |
| *lapB*_P1 | ACTTGGGGGCAACTATTGGG |
| *lapB*_P2 | AGATCGAGTTGGTGGCGAAG |
| *rpsL*_P1 | TATACACCACCACGCCGAAA |
| *rpsL*_P2 | TCACCACCGATGTACGAGGA |
| *recA*_P1 | GCCAACTGCCTGGTCATCTT |
| *recA*_P2 | GGCGTAGAACTTCAGTGCGT |
